# Macrocyclic Oxindole Peptide Epoxyketones—A Comparative Study of Macrocyclic Inhibitors of the 20S Proteasome

**DOI:** 10.1021/acsmedchemlett.4c00017

**Published:** 2024-03-27

**Authors:** Marion G. Götz, Kacey Godwin, Rachel Price, Robert Dorn, Gabriel Merrill-Steskal, William Klemmer, Hunter Hansen, Gautam Produturi, Megan Rocha, Mathias Palmer, Lea Molacek, Zack Strater, Michael Groll

**Affiliations:** † Department of Chemistry, Whitman College, Walla Walla, Washington 99362, United States; ‡ Technical University of Munich, TUM School of Natural Sciences, Department of Bioscience, Center for Protein Assemblies (CPA), Ernst-Otto-Fischer Strasse 8, 85748 Garching, Germany

**Keywords:** Proteasome, Inhibitor, Peptide macrocycle, Epoxyketone, Multiple myeloma, Carfilzomib

## Abstract

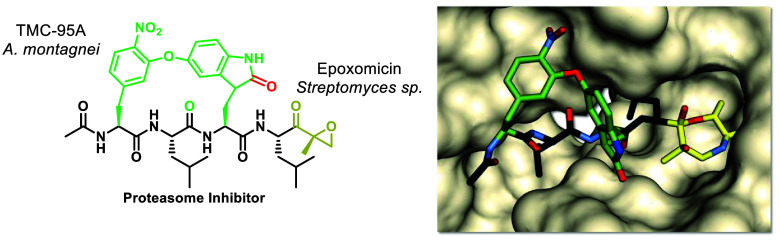

Peptide macrocycles have recently gained attention as protease inhibitors due to their metabolic stability and specificity. However, the development of peptide macrocycles with improved binding potency has so far been challenging. Here we present macrocyclic peptides derived from the clinically applied proteasome inhibitor carfilzomib with an oxindole group that mimics the natural product TMC-95A. Fluorescence kinetic activity assays reveal a high potency of the oxindole group (IC_50_ = 0.19 μM) compared with agents lacking this motif. X-ray structures of the ligands with the β5-subunit of the yeast 20S proteasome illustrate that the installed macrocycle forces strong hydrogen bonding of the oxindole group with β5-Gly23NH. Thus, the binding of our designed oxindole epoxyketones is entropically and enthalpically favored in contrast to more flexible proteasome inhibitors such as carfilzomib.

The 20S proteasome core particle (CP) is the major player in nonlysosomal protein degradation and mediates hydrolysis of most intracellular proteins to oligopeptides.^1^ This multifunctional protease complex is a barrel with four rings, two outer α-rings and two inner β-rings, each consisting of seven subunits.^2,3^ The active sites are located in the β1, β2, and β5 subunits and exhibit caspase-like or postglutamyl peptide hydrolase-like (PGPH-L), trypsin-like (T-L), and chymotrypsin-like (ChT-L) activities, respectively. Substrate hydrolysis is initiated through the N-terminal Thr1O^γ^. Blocking ChT-L activity leads to rapid accumulation of cellular proteins that can affect irreversible processes such as cell differentiation, cell cycle progression, and immune or stress response.^4−6^ Because of its central functional role, the CP represents an attractive drug target that has been studied in detail over the past decade.^7^ Peptide boron compounds such as bortezomib and ixazomib are prominent proteasome inhibitors (PI) applied in clinical settings for the treatment of multiple myeloma (MM), a lymphoproliferative B-cell tumor disease.^8−10^ While ixazomib has increased oral bioavailability, both blockbusters result in considerable side effects including peripheral neuropathy, anemia, and gastrointestinal complications due to their affinity for enzymes with an active site serine residue.^11^ These unwanted effects encouraged academia and the pharmaceutical industry to develop new but equipotent PIs with less off-target activity. One of the most promising drug candidates is carfilzomib, a tetrapeptide derivative of the natural product epoxomicin.^12^ Like boron compounds, carfilzomib induces cell death in a number of tumor cell lines and has been used since 2012 to treat MM and investigated in clinical trials to combat solid tumors.^13^ Notably, the α′,β′-epoxyketone electrophilic trap of this agent results in higher specificity for the CP. It irreversibly inhibits the proteasome by forming a cyclic adduct with the catalytic Thr1.^14,15^ This unique mode of action is based on the free N-terminal Thr1 amine, which increases the selectivity for epoxyketones. Nevertheless, carfilzomib has also been linked to severe off-target effects such as cardiac failure.^16,17^ Ultimately, the propensity of carfilzomib to kill nontumor cells and the resulting toxicities frequently require a reduction of the dosage or termination of the therapy.^17^ Therefore, the development of improved PIs is in great demand.

Recent interest in the pursuit of peptide macrocycles as protease inhibitors is based on several advantages. For example, the rigid scaffold of the macrocycle exhibits less off-target binding, increased stability, and improved membrane permeability.^18−20^ However, the main rationale for incorporating a macrocycle into a small-molecule inhibitor is that the reduced flexibility avoids a loss of entropy when binding to the target enzyme. On the other hand, the conformational rigidity may prevent the ligand from forming desired interactions with the enzyme substructures, resulting in lower efficacy. For example, we have previously reported that linear peptide aldehydes are two times more potent for the proteasome than tetrapeptide **16** cyclized with a biphenyl ether bridge (Figure 1).^21^ Nevertheless, the macrocyclic aldehydes still inhibit the proteasome at the cellular level and have 550-fold selectivity for the CP compared to that of cathepsin B, whereas the linear variant displays only 80-fold selectivity. Macrocyclic peptides have also been shown to inhibit parasitic proteasomes,^22,23^ and Li et al. have recently reported submicromolar inhibition of macrocyclic peptide epoxyketones that are cyclized via an aliphatic linker.^24^

**Figure 1:**
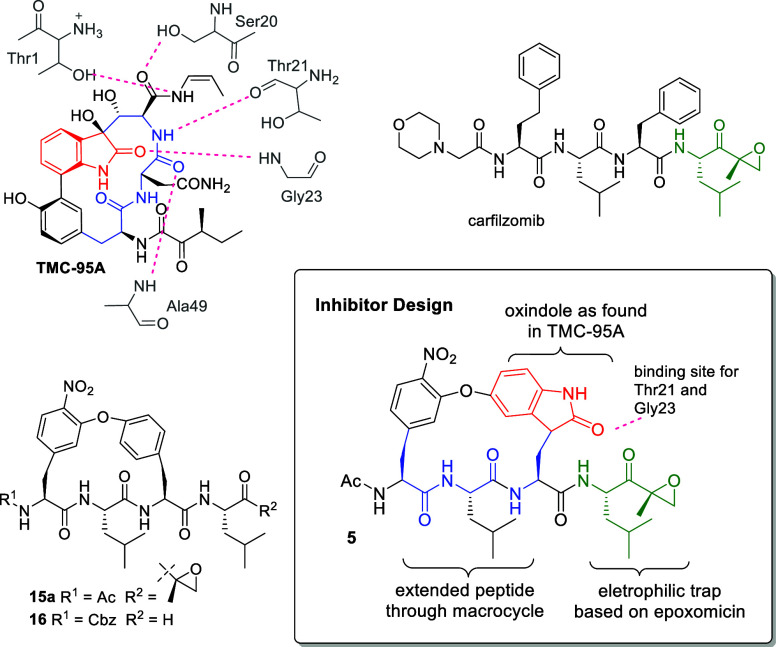
Design of the macrocyclic peptide epoxyketone **5** inspired by the electrophilic epoxyketone trap in epoxomicin and carfilzomib as well as the oxindole-containing macrocycle of TMC-95A, both derived from natural products, and considering previous findings on the macrocyclic peptide aldehyde **16**.^21^

The aim of our current study is to compensate for the loss of macrocycle potency as observed in previously developed macrocyclic peptide aldehydes^21^ by introducing additional binding sites into the macrocycle that interact with residues of subunit β5. As a blueprint, we used TMC-95A (Figure 1), a natural cyclic peptide metabolite from the fungus *Apiospora montagnei*, which is a noncovalent potent and selective CP inhibitor.^25^ Its macrocycle contains an oxindole moiety that offers an additional hydrogen bonding element between the CO of the oxidized tryptophan and β5Gly23NH.^26−30^ Replacing the tyrosine P2 amino acid residue of the biphenyl ether design in the previously reported tetrapeptide aldehydes, such as aldehyde **16**, with a hydroxylated oxindole derived from hydroxytryptophan introduces the desired H-bond donor carbonyl, similar to the macrocycle of TMC-95A.^21^ In order to obtain preliminary evidence that would support our rationale, we generated low-energy conformations by applying MOE (Molecular Operating Environment) and MMFF (Merck molecular force field) parameters. Using the Amber12:EHT (Assisted Model Building with Energy Refinement: Extended Hückel Theory) force field and the crystal structure of the human constitutive proteasome in complex with carfilzomib,^31^ we calculated the minimum-energy conformation of the β4β5 dimer bound to macrocyclic analogues. When comparing yeast proteasome bound macrocyclic biphenyl ethers^32,33^ with the oxindole modified design, two additional H-bonds between the oxindole and Thr21OH^γ^ and Gly23NH are predicted in the proteasome active site. Furthermore, we decided to replace the C-terminal aldehyde electrophilic trap with a C-terminal epoxyketone analogous to carfilzomib. The tetrapeptide carfilzomib contains Leu residues in its P1 and P3 positions in order to target the S1 and S3 pockets, respectively, of the ChT-L site.^34−36^ We also selected Leu residues for these positions, where the P3 Leu was integrated in the macrocyclic design similar to MG-132,^37,38^ the standard for proteasome inhibition assays, and carfilzomib resulting in the target peptide macrocyclic epoxyketone **5**.

Synthesis (for details, see the Supporting Information) of the macrocyclic oxindole peptides followed the general approach of preparation of a linear peptide that was subjected to macrocyclization by intramolecular S_N_Ar (Scheme 1).^39^ The non-natural phenylalanine analogue Ac-Phe(3-F,4-NO_2_)-OH functionalized with the strong electron-withdrawing nitro and fluorine substituents essential for S_N_Ar was synthesized as previously reported and first described by Vergne et al.^40^ The linear peptide **3** was derived from commercially available hydroxylated Trp **1** (5-HTP) by protective esterification with subsequent coupling of the peptide to Boc-Leu-OH and Boc deprotection. Coupling the resulting dipeptide **2** to the non-natural acetylated Phe derivative to produce tripeptide **3**, however, was less straightforward than anticipated. Using standard HOBt/EDC chemistry under basic conditions produced a large quantity of the *O*-acylated side product, where the aromatic hydroxyl of the 5-HTP residue was found to be acylated rather than the Leu amine. For improved yields, future preparation of peptides containing hydroxylated indoles may require orthogonal protection. Macrocyclization of tripeptide **3** was then pursued using S_N_Ar under basic conditions developed by Boger et al.^41^ We have previously successfully used the same conditions for the macrocyclization of nucleophilic Tyr with the Cbz-Phe(3-F,4-NO_2_) residue in cyclic peptide aldehydes.^21^ While cyclization with Tyr required 2–3 days, S_N_Ar by 5-HTP produced maximum yields of 53% in 2 weeks, likely due to the increased kinetic barrier. Several optimizations were explored, including a variety of bases, crown ether as an additive, temperature, and reactant concentration, but were not able to improve yields. In addition, the N-terminal Cbz we have previously used for protection, was not stable over the extended cyclization period. While Boc-protection survived the cyclization, the acidic conditions of the subsequent indole oxidation were not compatible. N-terminal acetyl protection was stable to both cyclization and oxidation conditions. Indole oxidation of the macrocycle by DMSO/HCl was more successful after carboxyl deprotection by saponification of the methyl ester. The C-terminal epoxyketone was prepared analogous to epoxyketone synthesis for carfilzomib by conversion of Boc-Leu-OH to the Weinreb amide suitable for Grignard alkylation with isopropenyl magnesium bromide (Scheme 2).^42^ Epoxidation with sodium hypochlorite resulted in a ratio of 2-(*R*)-epoxide to 2-(S)-epoxide of 2.1:1 and was followed by Boc-deprotection to produce diastereomers **8a** and **8b**. The preparation of other analogues required for comparing inhibitory potency involved a similar approach as the synthesis of macrocyclic epoxyketone **5**.

**Scheme 1:**
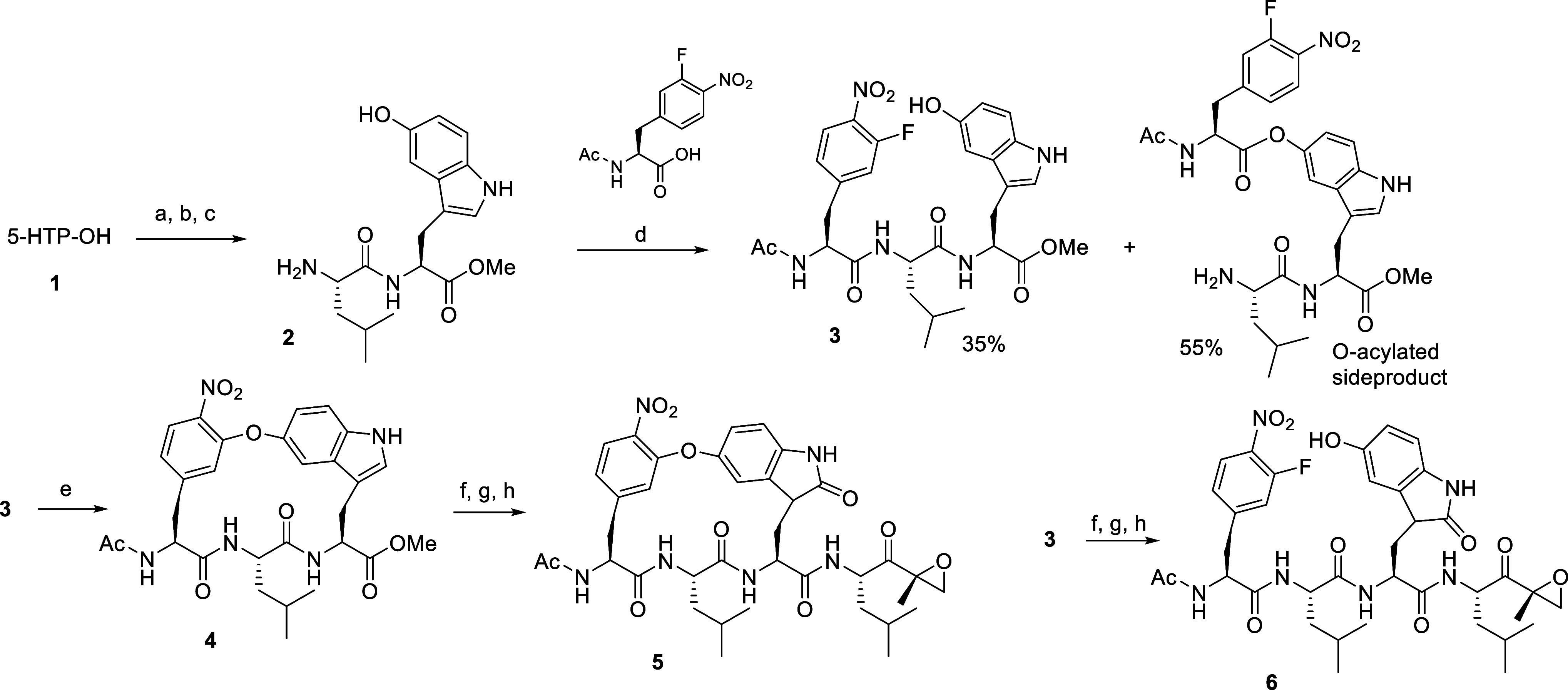
Synthesis of Macrocyclic and Linear Peptide Epoxyketones **5** and **6** Reagents and conditions: (a) SOCl_2_, MeOH, −20 °C, 3 h. (b) Boc-Leu-OH, EDC, HOBt, DIPEA, DMF, rt, 4 h. (c) 1:3 TFA/CH_2_Cl_2_ 0 °C, 2 h. (d) Ac-Phe(3-F,4-NO_2_)-OH, EDC, HOBt, DIPEA, DMF, rt, 4 h. (e) Molecular sieves (3 Å), K_2_CO_3_, CaCO_3_, DMF, 45 °C, 2 weeks. (f) MeOH, NaOH, rt, 8 h. (g) DMSO, phenol, HCl, AcOH, rt, 2 h. (h) **8a**, EDC, HOBt, DIPEA, rt, 4 h.

**Scheme 2:**
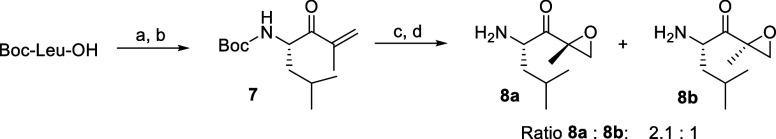
Synthesis of Epoxyketones **8a** and **8b** Reagents and conditions: (a) HN(OCH_3_)CH_3_·HCl, EDC, HOBt, DIPEA, DMF, rt, 4 h. (b) Isopropenyl MgBr, THF, 0 °C, 6 h. (c) 12.5% NaOCl, pyridine, −5 °C, 2 h. (d) 1:3 TFA/CH_2_Cl_2_ 0 °C, 2 h.

Macrocyclization was omitted for the linear tetrapeptide **6**, but other synthetic steps and conditions were analogous (Scheme 1). In order to compare macrocyclic inhibitors containing the C-terminal electrophilic epoxyketone to inhibitors lacking the electrophilic trap, analogue **12** with C-terminal amide was prepared as a tetrapeptide prior to cyclization and oxidation (Scheme 3). The C-terminal amide was synthesized by amide coupling of Boc-Leu-OH with the HOBt ammonium salt. Boc-protection was in this instance stable to the prolonged macrocyclization but replaced with Ac protection to make the comparison possible. Substitution of the oxindole with phenyl was achieved by replacing 5-HTP with either Tyr or homoTyr to furnish macrocycles **15a** and **15b** lacking the strategic oxindole and varying in macrocyclic ring size (Scheme 4). Both cyclizations required 2–3 days rather than 2 weeks as for the 5-HTP.

**Scheme 3:**
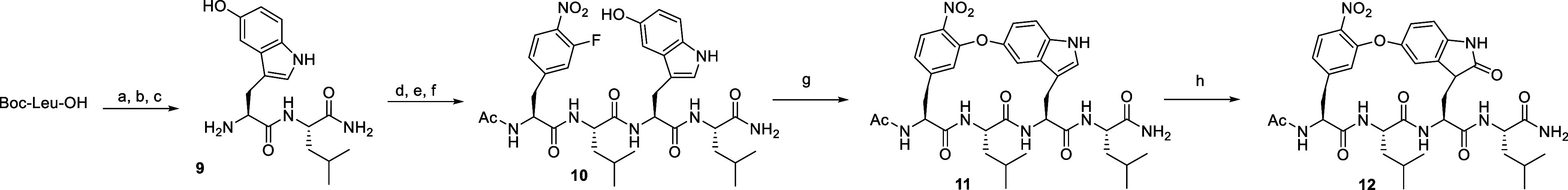
Synthesis of Macrocyclic Peptide Amide **12** Reagents and conditions: (a) HOBt-NH_3_, EDC, DIPEA, DMF, rt, 24 h. (b) (i) 1:3 TFA/CH_2_Cl_2_, 0 °C, 2 h. (ii) Boc-5-HTP-OH, EDC, HOBt, DIPEA, DMF, rt, 4 h. (c) 1:3 TFA/CH_2_Cl_2_, 0 °C, 2 h. (d) Boc-Leu-OH, EDC, HOBt, DIPEA, DMF, rt, 4 h. (e) 1:3 TFA/CH_2_Cl_2_, 0 °C, 2 h. (f) Ac-Phe(3-F,4-NO_2_)-OH, EDC, HOBt, DIPEA, DMF, rt, 8 h. (g) Molecular sieves (3 Å), K_2_CO_3_, CaCO_3_, DMF, 45 °C, 2 weeks. (h) DMSO, HCl, Phenol, AcOH, rt, 4 h.

**Scheme 4:**
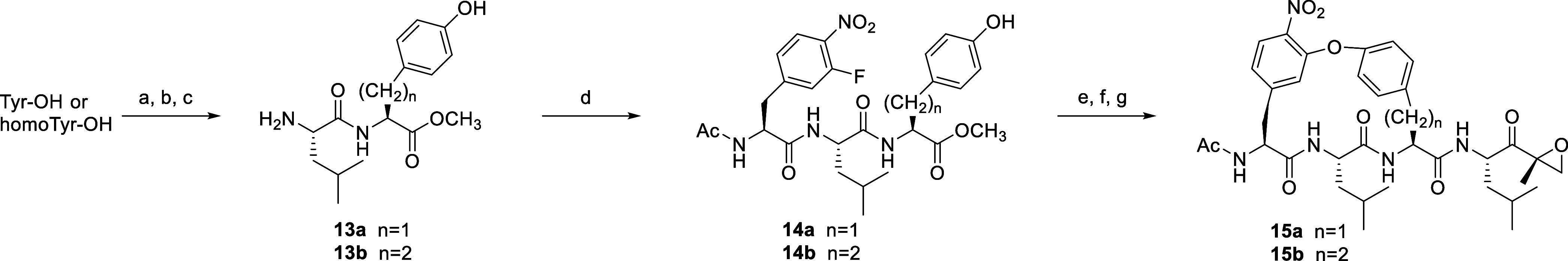
Synthesis of Macrocyclic Biphenyl Ether Epoxyketones **15a** and **15b** Reagents and conditions: (a) SOCl_2_, MeOH, −20 °C, 3 h. (b) Boc-Leu-OH, EDC, HOBt, DIPEA, DMF, rt, 4 h. (c) 1:3 TFA/CH_2_Cl_2_, 0 °C, 2 h. (d) Ac-Phe(3-F,4-NO_2_)-OH, EDC, HOBt, DIPEA, DMF, rt, 4 h. (e) Molecular sieves (3 Å), K_2_CO_3_, CaCO_3_, DMF, 45 °C, 3 days. (f) MeOH, NaOH, rt, 8 h. (g) **8a**, EDC, HOBt, DIPEA, rt, 4 h.

Fluorometric kinetic enzyme inhibition assays (for details, see Supporting Information) were performed for the ChT-L activity of the constitutive 20S proteasome to compare IC_50_ values for **5** with linear analogue **6**, unoxidized macrocycles **15a** and **15b**, and macrocyclic C-terminal amide **12** (Table 1). Furthermore, inhibition of the 20S proteasomal ChT-L activity was compared with T-L and PGPH-L activities and with inhibition of serine protease chymotrypsin and cysteine proteases cathepsin B and m-calpain. The lower IC_50_ value for oxindole-phenyl ether **5** (0.19 ± 0.02 μM) over the biphenyl ether analogues **15a** (0.55 ± 0.09 μM) and **15b** (2.90 ± 0.42 μM) confirms our hypothesis that introduction of an additional H-bonding element into the macrocyclic design improves potency. The diminished inhibition of **15b** over **15a** is likely due to the larger ring size of the more flexible macrocycle. The peptide backbone is no longer locked into an extended conformation, and the inhibitor experiences loss of entropy upon binding. The 4-fold lower potency of the linear analogue **6** (1.0 ± 0.10 μM) suggests that its energetically more favorable conformer does not place the oxindole carbonyl within H-bonding distance. The restricted conformation of the macrocycle, however, is able to direct the orientation of the oxindole carbonyl toward H-bonding interaction with the active site. In addition, the improved inhibitory potency can also be attributed to the reduced entropic penalty due to the less flexible macrocycle. Based on the placement of Leu residues in P1 and P3, the macrocyclic epoxyketone **5** does not significantly inhibit PGPH-L and T-L activity. While the inhibitory potency for the proteasome of **5** (0.19 ± 0.02 μM) and macrocyclic aldehyde **16** (0.24 ± 0.06 μM) is similar, their selectivity varies when considering other proteases. Epoxyketone **5** was not found to have any cross-reactivity with other representative proteases, whereas aldehyde **16** inhibits cathepsin B and m-calpain. Macrocyclic oxindole **12** was determined to be the least potent inhibitor as the presence of a C-terminal electrophilic trap is necessary for enhanced binding. No inhibition was observed of the serine protease chymotrypsin for any of the compounds.

**Table 1:** Kinetic Enzyme Assays for 20S Proteasome Activities and Other Proteases

	IC_50_ in μM					
	20S Proteasome					
Inhibitor	ChT-L Activity^a^	T-L Activity^a^	PGPH-L Activity^a^	Chymotrypsin^a^	Cathepsin B^a^	m-Calpain^a^
**5**	0.19 ± 0.02	N.I.^b^	52.5 ± 16.6	N.I.^b^	N.I.^b^	N.I.^b^
**6**	1.0 ± 0.10	N.I.^b^	N.I.^b^	N.I.^b^	N.I.^b^	N.I.^b^
**12**	25 ± 1.0	46.1 ± 3.1	N.I.^b^	N.I.^b^	N.I.^b^	N.I.^b^
**15a**	0.55 ± 0.09	N.I.^b^	N.I.^b^	N.I.^b^	N.I.^b^	N.I.^b^
**15b**	2.90 ± 0.42	N.I.^b^	N.I.^b^	N.I.^b^	N.I.^b^	N.I.^b^
**16**	0.24 ± 0.06	N.I.^b^	7.51 ± 2.32	N.I.^b^	3.1 ± 1.0	2.0 ± 0.3
carfilzomib	0.017 ± 0.012	N.I.^b^	1.05 ± 0.44	N.I.^b^	N.I.^b^	N.I.^b^

a Assay protocols are provided in the Supporting Information.

b No inhibition at 130 μM inhibitor concentration.

Crystal structures were obtained for macrocyclic oxindole epoxyketone **5** (PDB ID 8RHJ), linear oxindole epoxyketone **6** (PDB ID 8RHK), and biphenyl macrocyclic epoxyketone **15a** (PDB ID 8RHL) bound to yeast CP (Table S1, for details, see the Supporting Information). All three epoxides are bivalently bound to active site Thr1 via a 7-membered ring adduct (Figure 2).^15^ The alkoxide group of the generated hemiketal is stabilized by the oxyanion hole Gly47NH. As predicted by initial computational models, the crystal structure confirms that the oxindole carbonyl of **5** is H-bonded to Thr21OH^γ^ and Gly23NH, while **6** and **15a** lack these interactions. The structural superposition of **5** with **15a** reveals that the biphenyl-ether clamp is deficient in stabilizing H-bonds in the specificity pockets, resulting in reduced proteasomal affinity (Figure 2e). A structural overlay of the linear **5** and **6** demonstrates that the aromatic side chains of the P2 and P4 residues of the linear epoxide are rotated by 90° compared to those of the macrocycle, thereby placing the hydrogen-bonding carbonyl out of reach for additional active site interactions (Figure 2d). Moreover, for macrocyclic oxindole **5**, the flexibility of P2 and P4 residues is significantly restricted, causing additional entropic advantage upon binding as compared to **6**. Oxidation of indoles to oxindoles via DMSO/HCl is fairly well established;^43^ nevertheless, the potential for an enol/keto equilibrium can lead to inversion of the asymmetric oxindole sp^3^ carbon. Strikingly, MM2 (Molecular Mechanics) calculations reveal that the *R*-isomer of the oxindole moiety in **5** has a steric energy that is 12 kcal/mol lower than that of its *S*-isomer. These predictions are consistent with our crystallographic analyses, which confirm that the predominant isomer bound to the active site is the *R*-isomer of the oxindole, while the *S*-isomer is unable to form equivalent hydrogen bonding interactions. Furthermore, the COSY NMR analysis (see Supporting Information) verifies the oxindole sp^3^ C–H configuration and, in combination with LC (liquid chromatography)-analysis, validates a single isomer.

**Figure 2:**
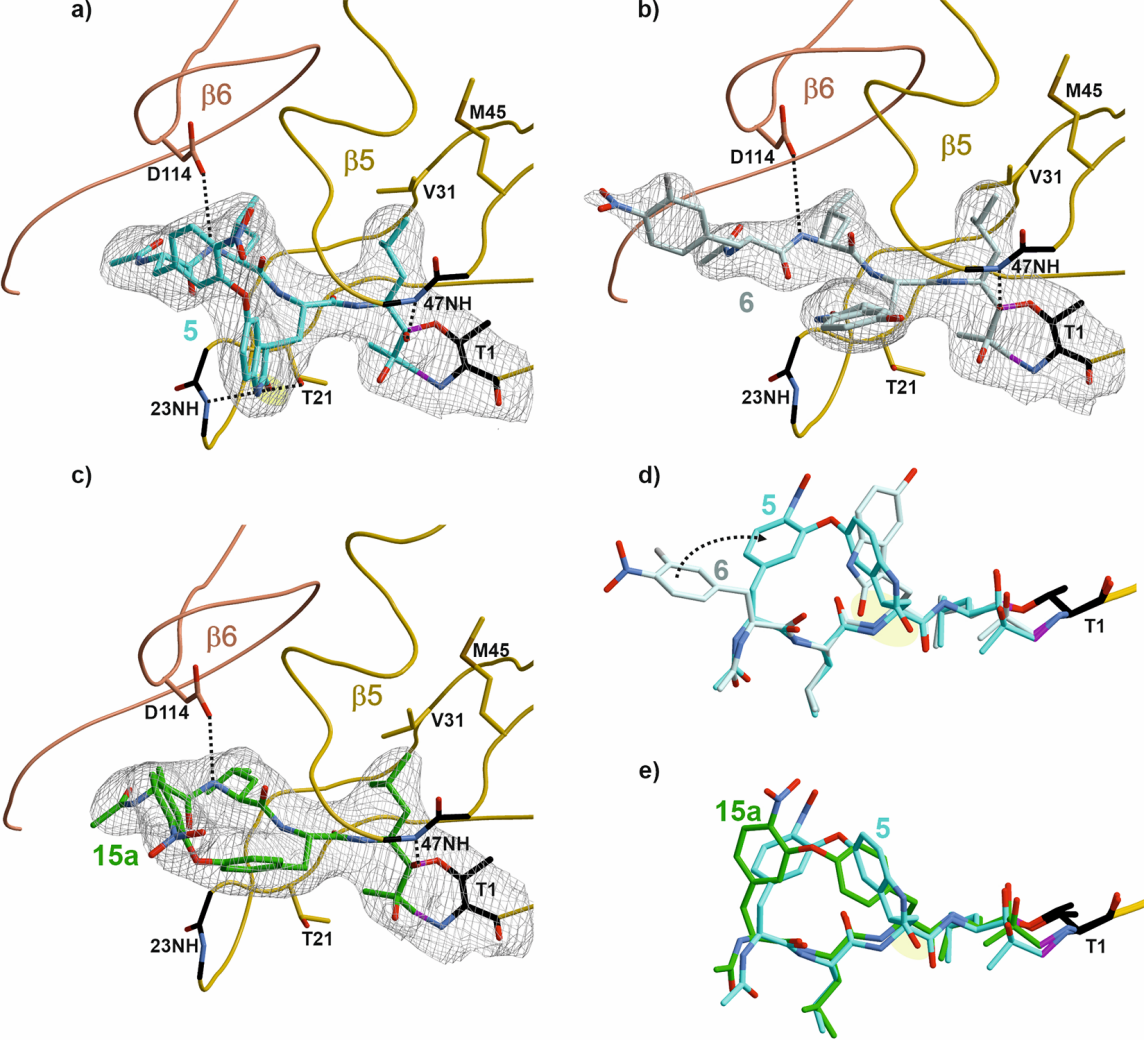
An intramolecular oxindole-phenyl clamp has a high affinity for proteasome inhibition. (a–c) The 2F_o_–F_c_ electron density map (gray mesh, contoured at 1σ) depicts **5** (cyan), **6** (gray), and **15a** (green) bound to the active-site nucleophile Thr1 (black) of subunit β5. The structural overlays of (d) **5** (cyan) and **6** (gray) as well as (e) **5** and **15a** reveal conformational differences of the respective ligands and provide molecular insights to the distinct IC_50_ values. The oxindole residue in **5** plays a major role in high-affinity binding to the proteasome and is highlighted by a yellow background.

In summary, incorporation of macrocycles in peptidyl inhibitors that reduce the entropic penalty upon binding and simultaneously introduce new H-bonding elements are potent and selective noncovalent or covalent proteasome inhibitors. The preparation of macrocyclic peptides, such as TMC-95A, is synthetically challenging, particularly due to the biaryl oxindole and the two asymmetric hydroxyl groups in the natural product, which are components of the macrocyclic clamp.^44^ Omission of both hydroxyl groups and modification of the oxindol-phenyl to a biaryl ether clamp make a more facile synthesis possible, particularly if an alternate protecting group strategy that avoids acylation side products is employed. Thus, these inhibitors are promising biaryl macrocyclic peptides with a C-terminal electrophilic epoxyketone that exhibit exceptional specificity for the proteasome, paving the way for numerous applications in drug development.

## Supporting Information Available

Synthetic procedures, compound characterization, ^1^H, ^13^C NMR, and HRMS spectra of all final compounds, enzyme assay protocols, crystallographic data (PDF)

## ABBREVIATIONS

Ac_2_O: acetic anhydride
Boc: *tert*-butyloxycarbonyl
Cbz: benzyloxycarbonyl
ChT-L: chymotrypsin-like
CP: core particle
PI: proteasome inhibitor
DIPEA: ethylenediisopropylamine
DMF: dimethylformamide
DMSO: dimethyl sulfoxide
EDC: 1-ethyl-3-(3-(dimethylamino)propyl)carbodiimide
EHT: extended Hückel theory
HOBt: hydroxybenzotriazole
HTP: hydroxytryptophan
LC: liquid chromatography
MM: multiple myeloma
MM2: molecular mechanics
MMFF: Merck molecular force field
MOE: Molecular Operating Environment
and PGPH: postglutamyl peptide hydrolase
S_N_Ar: nucleophilic aromatic substitution
T-L: trypsin-like
TFA: trifluoroacetic acid
