# Supplementary material for: Macrocyclic Oxindole Peptide Epoxyketones—A Comparative Study of Macrocyclic Inhibitors of the 20S Proteasome
Source: ACS Med Chem Lett. 2024 Mar 27;15(4):533–9. doi: 10.1021/acsmedchemlett.4c00017 (PMC11017298; doi:10.1021/acsmedchemlett.4c00017)
Supplement: Supplementary file 1 — ml4c00017_si_001.pdf [file ml4c00017_si_001.pdf]

Supplemental Information

Macrocyclic Oxindole Peptide Epoxyketones – A Comparative Study of Macrocyclic Inhibitors of the 20S Proteasome

Marion G. Götz,*,† Kacey Godwin,† Rachel Price,† Robert Dorn,† Gabriel Merrill-Steskal,† William Klemmer,† Hunter Hansen,† Gautam Produturi,† Megan Rocha,† Mathias Palmer,† Lea Molacek,† Zack Strater,† and Michael Groll*,‡

†Department of Chemistry, Whitman College, Walla Walla, WA 99362, USA
‡ Technical University of Munich, TUM School of Natural Sciences, Department of Bioscience, Center for Protein Assemblies, Ernst-Otto-Fischer Strasse 8, 85748 Garching, Germany

*Corresponding authors: gotzmg@whitman.edu, michael.groll@tum.de

Table of Contents

1. General methods S2
2. Experimental procedures for the preparation of **5**, **6**, **12**, **15a** and **15b** S3
3. NMR spectra of **5**, **6**, **12**, **15a** and **15b** S24
4. HRMS analysis of final compounds S29
5. COSY and LCMS spectra for macrocyclic epoxyketone **5** S31
6. *In Vitro* kinetic enzyme assays S32
7. Crystallographic data collection S34
8. References S36

1. General Methods

Peptide coupling reagents, amino acid derivatives, and solvents were purchased from VWR International. The starting material 3-fluoro-4-nitrotoluene was purchased from Matrix Scientific. Acylase I and isopropenyl magnesium bromide were purchased from Sigma-Aldrich. Materials for column chromatography were purchased from VWR International and TLC silica plates from EMD Chemicals. All NMR spectra were collected on a Bruker Avance III 400 MHz Ultrashield Plus Spectrometer. Low resolution mass analysis was obtained using an Agilent Technologies 1220 and 6120 Quadropole LC/MS with a linear gradient 0-100% AcCN in water over 16 minutes. High resolution mass spectral analysis was performed on an LTQ-Orbitrap with ESI source at the Chemistry Mass Spectrometry and Spectroscopy Facility at the University of Washington. 20S human RBC proteasome was purchased from South Bay Bio and bovine pancreatic α-chymotrypsin was purchased from Sigma Aldrich. Human liver cathepsin B and porcine erythrocytes calpain-1 were purchased from Millipore. All fluorogenic substrates were purchased from Millipore and bortezomib and carfilzomib were purchased from Advanced ChemBlocks Inc. Fluorescence measurements were conducted on a BioTekFlx800 plate reader on 96 well plates. Inhibitory constants were calculated using GraphPad Prism.

Safety statement. No unexpected or unusually high safety hazards were encountered.

2. Synthetic Procedures

2.1 Preparation of non-natural phenylalanine analog (*S*)-Phe(3-F,4-NO_2)-OH

Scheme 1S. Synthesis of non-natural phenylalanine not included in schemes, with minor modifications from previously described procedure.[1]

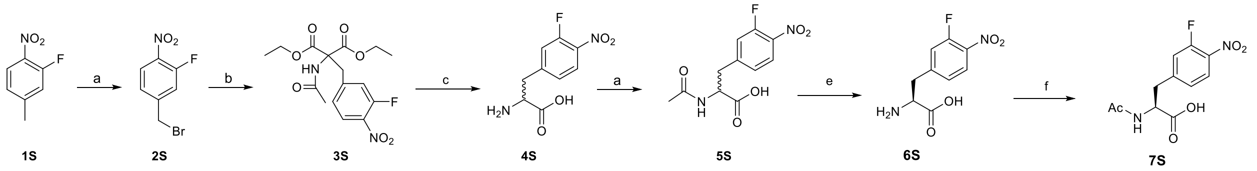

[a]Reagents and conditions: (a) NBS, AIBN, dimethyl carbonate, reflux, 20 h. (b) diethyl acetamidomalonate, NaH, DMF, rt, 4 h. (c) HCl (conc.), reflux, 24 h. (d) Ac_2O, $NaHCO_3$, dioxane/H_2O, rt, 12 h. (e) acylase I, pH 7.5, 37 °C, 20 h. (f) Ac_2O, $NaHCO_3$, dioxane/H_2O, rt, 12 h.

4-(Bromomethyl)-2-fluoro-1-nitrobenzene (2S). Commercially available 3-fluoro-4-nitrotoluene **1S** (16.96 g, 109.3 mmol), NBS (19.52 g, 109.3 mmol) and AIBN (0.30 g, 0.183 mmol) were dissolved in dimethyl carbonate (800 mL). The solution was then refluxed for 20 hours at 95 °C. Precipitates were removed by vacuum filtration and the solvent was removed under reduced pressure. The solids were dissolved in EtOAc (100 mL), and undissolved succinimide was filtered off and the solvent was removed under reduced pressure. This was repeated three times yielding a clear yellow-orange oil that was purified by column chromatography (7:1 petroleum ether/EtOAc, silica gel) yielding **2S** (14.72 g, 57.7%) as a yellow crystalline solid. ^1H NMR (400 MHz, $CDCl_3$) δ 8.06 (t, *J* = 8.2 Hz, 1H, arom-*H*), 7.34 (m, 2H, arom-*H*), 4.47 (s, 2H, CH_2Br).

Diethyl 2-acetamido-2-(3-fluoro-4-nitrobenzyl)malonate (3S): Diethyl acetamidomalonate (22.61 g, 104.1 mmol) was dissolved in anhydrous DMF (300 mL) and stirred at 0 °C . Solid NaH (60% in paraffin, 3.98 g, 99.4 mmol) was washed with pentane (50 mL), decanted, suspended in anhydrous DMF (100 mL), and then added slowly to the solution. 4-(Bromomethyl)-2-fluoro-1-nitrobenzene **2S** (22.14 g, 93.78 mmol) was added slowly to the reaction mixture resulting in a change of color to dark purple. The reaction mixture was stirred at room temperature for 4 hours by which time the solution turned a yellow color. The solvent was removed under reduced pressure and the solids were re-dissolved in EtOAc (200 mL). The product was precipitated with the addition of hexane and isolated by vacuum filtration yielding **3S** (29.67 g, 85.4%) as an off-white solid. ^1H NMR (400 MHz, DMSO-d_6) δ 8.29 (s, 1H, N*H*), 8.10 (t, *J* = 8.2 Hz, 1H, arom-*H*), 7.18

(dd, *J* = 12.3, 1.6 Hz, 1H, arom-*H*), 7.08 (dd, *J* = 8.4, 1.4 Hz, 1H, arom-*H*), 4.17 (q, *J* = 7.1 Hz, 4H, malonate-CH_2), 3.55 (s, 2H, Phe-CH_2), 1.96 (s, 3H, acyl-CH_3), 1.18 (t, *J* = 7.1 Hz, 6H, malonate-CH_3).

(*R,S*)-3-fluoro-4-nitrophenylalanine hydrochloride (**4S**): Diethyl 2-acetamido-2-(3-fluoro-4-nitrobenzyl)malonate **3S** (22.81 g, 61.59 mmol) was suspended in concentrated HCl (200 mL) and refluxed at 120 °C for 24 hours. The solution was filtered and the solvent was evaporated under reduced pressure and the resulting residue was further co-evaporated with 1:1 toluene/TBME (3 x 50 mL) to give **4S** as an off-white solid (14.04 g, 87.3 %). ^{1}H NMR (400 MHz, D_2O) δ 8.06 (d, *J* = 16.4 Hz, 1H, arom-*H*), 7.30 (dd, *J* = 12.1, 1.8 Hz, 1H, arom-*H*), 7.24 (dd, *J* = 8.5, 1.1 Hz, 1H, arom-*H*), 4.18 (dd, *J* = 7.5, 6.1 Hz, 1H, α-C*H),* 3.28 (m, 2H, Phe-CH_2).

(*R,S*)-*N*-acetyl-3-fluoro-4-nitrophenylalanine (5S): (*R,S*)-3-Fluoro-4-nitrophenylalanine hydrochloride **4S** (4.93 g, 18.6 mmol) was dissolved in a solution of H_2O and dioxane (1:1, 100 mL). The pH was adjusted to 8-9 with the addition of solid $NaHCO_3$. Acetic anhydride (4.97 mL, 55.9 mmol) was added and the pH re-adjusted to 8-9 with additional $NaHCO_3$. The solution was stirred at room temperature overnight. Solid $NaHCO_3$ was removed by filtration and dioxane was evaporated under reduced pressure. The pH of the solution was adjusted to 2-3 with concentrated HCl and the product was extracted with EtOAc (3 x 75 mL). The extracts were washed with brine (3 x 50 mL), dried ($MgSO_4$), and evaporated under reduced pressure to yield **5S** (4.66 g, 92.5 %) as a beige solid. ^{1}H NMR (400 MHz, DMSO-d_6) δ 8.24 (d, *J* = 8.2 Hz, 1H, N*H*), 8.09 (t, *J* = 8.3 Hz, 1H, arom-*H*), 7.47 (dd, *J* = 12.6, 1.6 Hz, 1H, arom-*H*), 7.31 (dd, *J* = 8.4, 1.4 Hz, 1H, arom-*H*), 4.50 (m, 1H, α-C*H*), 3.19 (dd, *J* = 13.8, 4.9 Hz, 1H, Phe-C*H*), 2.97 (dd, *J* = 13.8, 9.8 Hz, 1H, Phe-C*H*), 1.78 (s, 3H, CO-CH_3).

(*S*)-3-fluoro-4-nitrophenylalanine (6S): (*R,S*)-*N*-acetyl-3-fluoro-4-nitrophenylalanine **5S** (2.92 g, 10.8 mmol) was suspended in phosphate buffer (0.1 M KH_2PO_4/K_2HPO_4, pH 7.5, 125 mL), aqueous KOH (0.1 M, 108 mL) was added and the suspension was shaken vigorously. Acylase I (2.77 g) was added and the solution was shaken and then incubated at 37 °C overnight. Using Amicron filtration under argon pressure (cut off > 10 kDa) the acylase I enzyme was removed and the filtrate was acidified to pH 2-3 with concentrated HCl. The undesired *R*-isomer was extracted

with EtOAc (3 x 75 mL) and the remaining aqueous solution was neutralized to pH 7 with the addition of aqueous KOH (1 M). The solvent was evaporated under reduced pressure until a white solid started to form. Further crystallization was promoted by refrigeration and crystals were collected using vacuum filtration yielding **6S** (1.02 g, 83.0%) as a light-yellow solid. ^{1}H NMR (400 MHz, D_2O) δ 8.05 (t, *J* = 8.1 Hz, 1H, arom-*H*), 7.27 (dd, *J* = 12.2, 1.7 Hz, 1H, arom-*H*), 7.23 (dd, *J* = 8.5, 1.2 Hz, 1H, arom-*H*), 3.93 (dd, *J* = 14.6, 5.8 Hz, 1H, α-C*H*), 3.25 (dd, *J* = 14.6, 5.9 Hz, 1H, Phe-C*H*), 3.15 (dd, *J* = 14.5, 7.5 Hz, 1H, Phe-C*H*).

(*S*)-*N*-acetyl-3-fluoro-4-nitrophenylalanine (7S): (*S*)-3-fluoro-4-nitrophenylalanine **6S** (1.92 g, 8.40 mmol) was dissolved in a solution of H_2O and dioxane (1:1, 100 mL). The pH was adjusted to 8-9 with the addition of solid $NaHCO_3$. Acetic anhydride (2.24 mL, 21.2 mmol) was added and the pH re-adjusted to 8-9 with additional $NaHCO_3$. The solution was stirred overnight at room temperature. Using vacuum filtration, the $NaHCO_3$ was removed and dioxane was evaporated under reduced pressure. The solution was adjusted to pH 2-3 with concentrated HCl and the product was extracted with EtOAc (3 x 75 mL). The extracted solution was washed with brine (3 x 50 mL), dried ($MgSO_4$), and evaporated under reduced pressure to yield enantiomerically pure **7S** (1.64 g, 72.1 %) as a light-yellow solid. ^{1}H NMR (400 MHz, DMSO-d_6) δ 12.73 (s, broad, 1H, COOH), 8.26 (d, *J* = 8.2 Hz, 1H, N*H*), 8.09 (t, *J* = 8.2 Hz, 1H, arom-*H*), 7.47 (dd, *J* = 12.6, 1.6 Hz, 1H, arom-*H*), 7.32 (d, *J* = 1.4 Hz, 1H, arom-*H*), 4.52 (m, 1H, α-C*H*), 3.19 (dd, *J* = 13.8, 4.9 Hz, 1H, C*H*$_2$), 2.99 (dd, *J* = 9.9, 4.9 Hz, 1H, C*H*$_2$), 1.99 (s, 3H, CO-C*H*$_3$).

2.2 Preparation of oxindole and epoxyketone containing macrocycle 5

Scheme 2S. Preparation of oxindole and epoxyketone containing macrocycle 5.

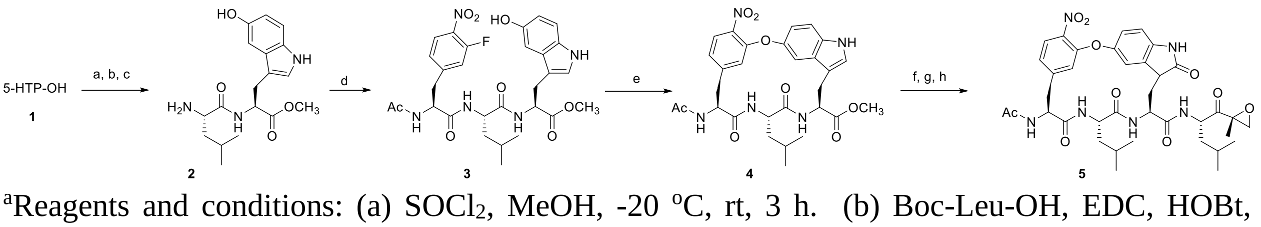

[a]Reagents and conditions: (a) $SOCl_2$, MeOH, -20 °C, rt, 3 h. (b) Boc-Leu-OH, EDC, HOBt, DIPEA, DMF, rt, 4 h. (c) 1:3 TFA/CH_2Cl_2, 0 °C, 2 h. (d) **7S**, EDC, HOBt, DIPEA, DMF, rt, 4 h. (e) molecular sieves (3 Å), K_2CO_3, $CaCO_3$, DMF, 45 °C, 2 weeks. (f) MeOH, NaOH, rt, 8 h. (g) DMSO, phenol, HCl, AcOH, rt, 2 h. (h) **8a**, EDC, HOBt, DIPEA, rt, 4 h.

5-HTP-OMe (Not numbered in schemes)**:** 5-HTP-OH **1** (8.00g, 36.3 mmol) was dissolved in MeOH (200 mL) and stirred for 10 minutes at -20 °C. Thionyl chloride (12.9 g, 110 mmol) was added dropwise to the solution and stirred for 3 hours at room temperature. The solvent was removed under reduced pressure yielding a brown syrup that was washed with EtOAc (100 mL) to give 5-HTP-OMe (9.28 g, 94.5% yield) as a white powder. ^{1}H NMR (400 MHz, DMSO-d$_6$) δ 10.78 (s, 1H, indole N*H*), 8.50 (s, broad, 3H, O*H* and N*H$_2$*), 7.16 (d, *J* = 8.6 Hz, 1H, arom-*H*), 7.14 (d, *J* = 2.3 Hz, 1H, arom-*H*), 6.78 (d, *J* = 2 Hz, 1H, arom-*H*), 6.63 (dd, *J* = 10.8, 6.4 Hz, 1H, arom-*H*), 4.18 (m, 1H, α-C*H*), 3.68 (s, 3H, OC*H$_3$*), 3.21, (d, *J* = 1.3 Hz, 1H, 5-HTP-C*H$_2$*), 3.19 (d, 1H, *J* = 2.3 Hz, 5-HTP-C*H$_2$*).

Boc-Leu-5-HTP-OMe (Not numbered in schemes)**:** 5-HTP-OMe (9.82 g, 36.3 mmol) followed by Boc-Leu-OH (9.05 g, 36.3 mmol), HOBt (6.75 g, 49.9 mmol), EDC (8.35 g, 43.6 mmol), and DIPEA (9.39 g, 72.6 mmol) were dissolved in DMF (300 mL) and stirred for 8 hours. The solvent was removed under reduced pressure and the residue dissolved in EtOAc (300 mL). The solution was washed with $KHSO_4$ (5%, 3 x 100 mL) and $NaHCO_3$ (5%, 3 x 100 mL), dried over anhydrous $MgSO_4$, and the solvent evaporated under reduced pressure. The crude product was purified by column chromatography (3.5% MeOH in DCM, silica gel), yielding Boc-Leu-5-HTP-OMe (11.2 g, 69.0% yield) as an off-white powder. ^{1}H NMR (400 MHz, DMSO-d$_6$) δ 10.56 (s, 1H, indole N*H*), 8.61 (s, 1H, indole O*H*), 8.07 (d, *J* = 7.3 Hz, 1H, N*H*), 7.11 (d, *J* = 8.6 Hz, 1H, arom-*H*), 6.87 (m, 1H, N*H*), 6.77 (d, *J* = 2.1 Hz, 1H, arom-*H*), 6.59 (dd, *J* = 8.6, 2.3 Hz, 1H, arom-*H*), 4.47 (q, *J* = 6.7 Hz, 1H, α-C*H*), 4.01 (m, 1H, α-C*H*), 3.56 (s, 3H, OC*H$_3$*), 3.01 (m, 2H, 5-HTP-C*H$_2$*), 1.54 (m, 1H, Leu-C*H*), 1.37 (s, 11H, Boc-C(C*H$_3$*)$_3$ and Leu-C*H$_2$*), 0.86 (m, 6H, 2 x Leu-C*H$_3$*).

TFA • Leu-5-HTP-OMe (2): Boc-Leu-5-HTP-OMe (9.39 g, 20.9 mmol) was dissolved in TFA/DCM (1:3, 150 mL) that had been cooled 0 °C and was stirred for 12 hours. The solvent was removed under reduced pressure and the remaining clear brown syrup triturated with *t*-butyl methyl ether/hexanes (2:1, 100 mL) at 0 °C, yielding TFA • Leu-5-HTP-OMe **2** (8.52 g, 88.4% yield), as a dark grey solid. ^{1}H NMR (400 MHz, DMSO-d$_6$) δ 10.61 (s, 1H, indole N*H*), 8.90 (d, *J* = 7.2 Hz, 1H, N*H*), 8.10 (s, broad, 3H, O*H* and N*H$_2$*), 7.14 (d, *J* = 8.6 Hz 1H, arom-*H*), 7.09 (d, *J* = 2.2 Hz, 1H, arom-*H*), 6.81 (d, *J* = 2.1 Hz, 1H, arom-*H*), 6.61 (dd, *J* = 8.6, 2.2 Hz, 1H, arom-*H*),

4.56 (m, 1H, α-C*H*), 3.79 (m, 1H, α-C*H*), 3.61 (s, 3H, OCH_3), 3.02 (m, 2H, 5-HTP-CH_2), 1.67 (m, 1H, Leu-C*H*), 1.56 (m, 2H, Leu-CH_2), 0.91 (m, 6H, 2 x Leu-CH_3).

Ac-Phe(3-F,4-NO$_2$)-Leu-5-HTP-OMe (3): TFA • Leu-5-HTP-OMe **2** (6.76 g, 14.7 mmol) followed by (*S*)-*N*-acetyl-3-fluoro-4-nitrophenylalanine **7S** (3.96 g, 14.7 mmol), HOBt (2.18 g, 16.1 mmol), EDC (3.37 g, 17.6 mmol), and DIPEA (3.78 g, 29.3 mmol) were dissolved in DMF (100 mL) and the reaction mixture was stirred at room temperature for 8 hours. The solvent was removed under reduced pressure, and the solids were re-dissolved in EtOAc and washed with $KHSO_4$ (5%, 3 x 50 mL), saturated $NaHCO_3$ (3 x 50 mL), and brine (2 x 50 mL). The solution was dried over anhydrous $MgSO_4$, and the solvent removed under reduced pressure. The product was purified by column chromatography (3.5% MeOH in DCM, silica gel), yielding Ac-Phe(3-F,4-NO$_2$)-Leu-5-HTP-OMe **3** (3.10 g, 35.3% yield) as an orange powder. ^{1}H NMR (400 MHz, DMSO-d$_6$) δ 10.56 (s, 1H, indole N*H*), 8.63 (s, 1H, indole O*H*), 8.32 (d, *J* = 7.0 Hz, 1H, N*H*), 8.11 (m, 2H, N*H* x 2), 8.05 (t, *J* = 8.2 Hz, 1H, arom-*H*), 7.44 (d, *J* = 12.3 Hz, 1H, arom-*H*), 7.28 (d, *J* = 8.6 Hz, 1H, arom-*H*), 7.10 (d, *J* = 8.6 Hz, 1H, arom-*H*), 7.06 (d, *J* = 1.8 Hz, 1H, arom-*H*), 6.78 (d, *J* = 1.8 Hz, 1H, arom-*H*), 6.57 (dd, *J* = 8.6, 2.1 Hz, 1H, arom-*H*), 4.60 (m, 1H, α-C*H*), 4.48 (q, *J* = 7.0 Hz, 1H, α-C*H*), 4.36 (q, *J* = 7.6 Hz, 1H, α-C*H*), 3.55 (s, 3H, OCH_3), 3.17 (d, *J* = 5.1 Hz, 1H, arom-C*H*), 3.02 (m, 2H, 2 x arom-C*H*), 2.80 (dd, *J* = 13.2, 10.2 Hz, 1H, arom-C*H*), 1.75 (s, 3H, CO-CH_3), 1.57 (m, 1H, Leu-C*H*), 1.45 (m, 2H, Leu-CH_2), 0.90 (d, *J* = 6.5 Hz, 3H, Leu-CH_3), 0.85 (d, *J* = 6.4 Hz, 3H, Leu-CH_3). Molecular formula $C_{29}H_{34}FN_5O_8$, ESI-MS calcd for 599.2, found 600.3 $[M+H]^+$.

Macrocycle Ac-Phe(3-F,-4-NO$_2$)-Leu-5-HTP-OMe (4): The linear peptide was cyclized following a method first reported by Boger et al.[2] Molecular sieves (3 Å, 10.0 g), K_2CO_3 (2.15 g, 15.5 mmol), and $CaCO_3$ (1.55 g, 15.5 mmol) were dried at 170 °C under vacuum for 5 hours. Ac-Phe(3-F,4-NO$_2$)-Leu-5-HTP-OMe **3** (3.10 g, 5.18 mmol) was dissolved in DMF (200 mL) and added to the dried reagents and stirred for 2 weeks at 45 °C. Cyclization was monitored using LC-MS. The solution was filtered over Celite and the solvent removed under reduced pressure. The crude product was purified via column chromatography (3.5% MeOH in DCM, silica gel), yielding macrocyclized Ac-Phe(3-F,4-NO$_2$)-Leu-5-HTP-OMe (890 mg, 26% yield) as a light yellow powder. ^{1}H NMR (400 MHz, DMSO-d$_6$) δ 11.15 (s, 1H, indole N*H*), 8.62 (d, *J* = 8.9 Hz, 1H, N*H*),

8.05 (d, *J* = 7.9 Hz, 1H, N*H*), 7.91 (d, *J* = 8.4 Hz, 1H, arom-*H*), 7.74 (d, *J* = 7.0 Hz, 1H, N*H*), 7.56 (s, 1H, arom-*H*), 7.37 (d, *J* = 8.7 Hz, 1H, arom-*H*), 7.34 (s, 1H, arom-*H*), 6.95 (d, *J* = 8.1 Hz, 1H, arom-*H*), 6.75 (d, *J* = 8.2 Hz, 1H, arom-*H*), 6.30 (s, 1H, arom-*H*), 4.68 (t, *J* = 8.9 Hz, 1H, α-C*H*), 4.59 (s, 1H, α-C*H*), 4.31 (d, *J* = 6.9 Hz, 1H, α-C*H*), 3.70 (s, 3H, OCH_3), 3.24 (d, *J* = 13.8 Hz, 1H, arom-C*H*), 3.04 (m, 1H, arom-C*H*), 2.91 (m, 1H, arom-C*H*), 2.65 (m, 1H, arom-C*H*), 1.89 (s, 3H, CO-CH_3), 1.51 (m, 1H, Leu-C*H*), 1.42 (m, 1H, Leu-C*H*), 1.31 (m, 1H, Leu-C*H*), 0.87 (d, *J* = 6.3 Hz, 3H, Leu-CH_3), 0.81 (d, *J* = 6.2 Hz, 3H, Leu-CH_3). Molecular formula $C_{29}H_{33}N_5O_8$, ESI-MS calcd for 579.2, found 580.2 $[M+H]^+$.

Macrocycle Ac-Phe(3-F,4-NO_2)-Leu-5-HTP-OH (Not numbered in schemes)**:** Macrocycle Ac-Phe(3-F,4-NO_2)-Leu-5-HTP-OMe **4** (600 mg, 1.03 mmol) was saponified by addition of NaOH (3.0 mL, 3.1 mmol) in MeOH (50 mL) and the reaction mixture was stirred at room temperature for 8 hours. The solvent was removed under reduced pressure and the solids re-dissolved in H_2O. The pH was adjusted to 2 with concentrated HCl and the product was extracted with EtOAc (3 x 50 mL). The solvent was evaporated under reduced pressure yielding macrocyclized Ac-Phe(3-F,4-NO_2)-Leu-5-HTP-OH (632 mg, 99% yield) as a brown solid. ^1H NMR (400 MHz, DMSO-d_6) δ 11.10 (s, 1H, indole N*H*), 8.48 (d, *J* = 9.0 Hz, 1H, N*H*), 8.00 (d, *J* = 8.2 Hz, 1H, N*H*), 7.91 (d, *J* = 8.4 Hz, 1H, arom-*H*), 7.74 (d, *J* = 7.1 Hz, 1H, N*H*), 7.53 (s, 1H, arom-*H*), 7.37 (d, *J* = 8.6 Hz, 1H, arom-*H*), 7.32 (s, 1H, arom-*H*), 6.95 (d, *J* = 8.4 Hz, 1H, arom-*H*), 6.74 (dd, *J* = 8.7, 2.1 Hz, 1H, arom-*H*), 6.31 (s, 1H, arom-*H*), 4.58 (m, 2H, 2 x α-C*H*), 4.31 (q, *J* = 5.8 Hz, 1H, α-C*H*), 3.23 (d, *J* = 15.2 Hz, 1H, arom-C*H*), 3.00 (dd, *J* = 14.8, 10.4 Hz, 1H, arom-C*H*), 2.89 (dd, *J* = 13.2, 6.4 Hz, 1H, arom-C*H*), 2.64 (dd, *J* = 13.3, 3.1 Hz, 1H, arom-C*H*), 1.89 (s, 3H, CO-CH_3), 1.52 (m, 1H, Leu-C*H*), 1.40 (m, 1H, Leu-C*H*), 1.31 (m, 1H, Leu-C*H*), 0.84 (d, *J* = 6.4 Hz, 3H, Leu-CH_3), 0.79 (d, *J* = 6.4 Hz, 3H, Leu-CH_3).

Macrocycle Ac-Phe(3-F,4-NO_2)-Leu-5-HTP(ox)-Leu-1-methyloxiranyl (5): Macrocyclized (3-Phe to 5-HTP aryl ether) Ac-Phe(3-F,4-NO_2)-Leu-5-HTP-OH (632 mg, 1.12 mmol) followed by phenol (2 drops), DMSO (219 mg, 2.80 mmol) and concentrated HCl (1 mL) were added to acetic acid (30 mL) and stirred at room temperature for 2 hours. The solvent was removed under reduced pressure and the residue was washed with EtOAc and centrifuged yielding oxidized (5-HTP oxindole) macrocyclized (3-Phe to 5-HTP aryl ether) Ac-Phe(3-F,4-NO_2)-Leu-5-HTP-OH (118

mg, 18% yield), as a crude grey powder. Molecular formula $C_{28}H_{31}N_5O_9$, ESI-MS calcd for 581.2, found 582.2 $[M+H]^+$. Oxidized Ac-Phe(3-F,4-NO_2)-Leu-5-HTP(ox)-OH (151 mg, 0.260 mmol) followed by EDC (60 mg, 0.31 mmol), HOBt (48 mg, 0.29 mmol), DIPEA (64 mg, 0.52 mmol), and TFA • Leu-1-methyloxiranyl **8a** (74.1 mg, 0.260 mmol) were dissolved in DMF and stirred for 8 hours. The solvent was removed under reduced pressure and the residue dissolved in EtOAc. The solution was washed with $KHSO_4$ (5%, 3 x 50 mL), saturated $NaHCO_3$ (5%, 3 x 50 mL) and brine (3 x 50 mL), then dried ($MgSO_4$). The solvent was evaporated under reduced pressure, and the crude solid was purified by column chromatography (5% MeOH in DCM, silica gel), yielding macrocycle Ac-Phe(3-F,4-NO_2)-Leu-5-HTP(ox)-Leu-methyloxiranyl **5** (35 mg, 19% yield) as a yellow solid. ^{1}H NMR (400 MHz, methanol-d$_4$) δ 8.47 (s, 1H, N*H*), 7.98 (d, *J* = 8.4 Hz, 1H, arom-*H*), 7.25 (d, *J* = 8.5 Hz, 1H, arom-*H*), 7.14 (d, *J* = 2.1 Hz, 1H, arom-*H*), 7.12 (d, *J* = 2.1 Hz, 1H, arom-*H*), 7.02 (m, 2H, 2 x arom-*H*), 4.80 (m, 2H, 2 x α-C*H*), 4.47 (m, 1H, α-C*H*), 4.31 (m, 1H, α-C*H*), 3.58 (dd, *J* = 12.7, 4.1 Hz, 1H, indole-C*H*), 3.12 (d, *J* = 4.9 Hz, 1H, epoxide-C*H*), 3.05 (m, 1H, arom-C*H*), 2.95 (d, *J* = 3.9 Hz, 1H, arom-C*H*), 2.92 (d, *J* = 5.0 Hz, 1H, epoxide-C*H*), 2.60 (m, 1H, arom-C*H*), 2.06 (s, 3H, CO-C*H*$_3$), 1.71 (m, 1H, arom-C*H*), 1.60 (m, 4H, 4 x Leu-C*H*), 1.44 (m, 4H, Leu-C*H* and epoxide-C*H*$_3$), 1.41 (m, 1H, Leu-C*H*), 0.87 (m, 12H, 4 x Leu-C*H*$_3$). ^{13}C NMR (101 MHz, methanol-d$_4$) δ 207.70, 179.30, 172.72, 172.48, 171.20, 170.98, 150.71, 150.30, 143.77, 138.87, 138.73, 128.62, 125.74, 122.90, 120.18, 119.45, 116.44. 110.74, 58.58, 53.99, 52.32, 51.62, 50.59, 49.40, 42.37, 40.82, 38.59, 38.20, 32.41, 24.76, 23.99. 22.25, 21.86, 21.47, 21.31, 19.94, 15.59. Molecular formula $C_{37}H_{46}N_6O_{10}$, HRMS calcd for $[M+H]^+$ 735.3363, found 735.3348 $[M+H]^+$.

2.3 <u>Synthesis of oxindole and epoxyketone containing linear epoxyketone 6</u>

Scheme 3S. Preparation of linear oxindole tetrapeptide epoxyketone 6.

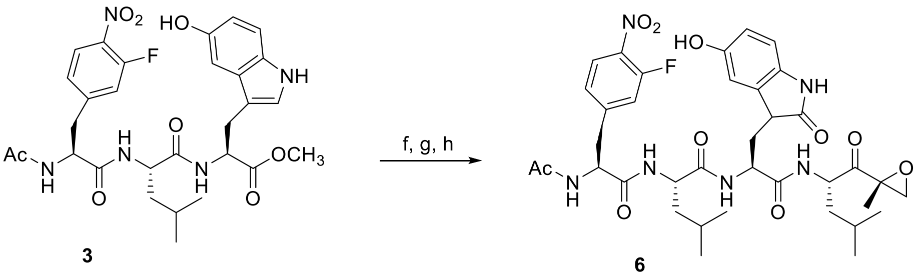

^{a}Reagents and conditions: (f) MeOH, NaOH, rt, 8 h. (g) DMSO, phenol, HCl, AcOH, rt, 2 h. (h) **8a** EDC, HOBt, DIPEA, rt, 4 h.

Ac-Phe(3-F,4-NO_2)-Leu-5-HTP-OH (Not numbered in scheme)**:** Ac-Phe(3-F,4-NO_2)-Leu-5-HTP-OMe **3** (2.73g, 4.55 mmol) was dissolved in MeOH (100 mL). NaOH (1M, 45 mL, 45.5 mmol) was added and the solution was stirred overnight. The solvent was removed under reduced pressure and the solids were re-dissolved in H_2O. The pH was adjusted to 2 with HCl (3 M) and the aqueous layer was extracted with EtOAc (3 x 100 mL). The organic layer was washed with brine (3 x 50 mL), dried ($MgSO_4$), and the solvent was removed under reduced pressure to yield Ac-Phe(3-F,4-NO_2)-Leu-5-HTP-OH (1.61 g, 61%) as a dark solid. ^{1}H NMR (400 MHz, DMSO-d_6) δ 10.53 (s, 1H, indole-N*H*), 8.61 (s, 1H, indole-O*H*), 8.12 (m, 3H, 3 x NH), 7.76 (d, *J* = 8.2 Hz, 1H, arom-*H*), 7.22 (d, *J* = 1.4 Hz, 1H, arom-*H*), 7.10 (d, *J* = 8.6 Hz, 1H, arom-*H*), 7.05 (d, *J* = 2.2 Hz, 1H, arom-*H*), 6.95 (dd, *J* = 8.3, 1.4 Hz, 1H, arom-*H*), 6.83 (d, *J* = 2.1 Hz, 1H, arom-*H*), 6.58 (dd, *J* = 8.6, 2.3 Hz, 1H, arom-*H*), 4.60 (m, 1H, α-C*H*), 4.43 (m, 1H, α-C*H*), 4.36 (m, 1H, α-C*H*), 2.89 (m, 3H, arom-CH_2 and arom-C*H*), 2.77 (dd, *J* = 13.6, 10.4 Hz, 1H, arom-C*H*), 1.90 (s, 3H, CO-CH_3), 1.57 (m, 1H, Leu-C*H*), 1.46 (m, 2H, Leu-CH_2), 0.89 (d, *J* = 6.5 Hz, 3H, Leu-CH_3), 0.85 (d, *J* = 6.2 Hz, 3H, Leu-CH_3).

Ac-Phe(3-F,4-NO_2)-Leu-5-HTP(ox)-Leu-1-methyloxiranyl (6): Ac-Phe(3-F,4-NO_2)-Leu-5-HTP-OH (878 mg, 1.50 mmol), phenol (2 drops), DMSO (0.293 g, 3.75 mmol), concentrated HCl (1.5 mL) and acetic acid (40 mL, 0.699 mmol) were combined and stirred for 2 hours at room temperature. Solvents were removed under reduced pressure and the solids were suspended in EtOAc. The suspension was centrifuged and the supernatant decanted yielding crude oxidized Ac-Phe(3-F,4-NO_2)-Leu-5-HTP(ox)-OH (522 mg, 58% crude yield). Crude oxidized Ac-Phe(3-F,4-NO_2)-Leu-5-HTP-OH (423 mg, 0.703 mmol) was dissolved in DMF and TFA • Leu-1-methyloxiranyl **8a** (201 mg, 0.703 mmol), EDC (162 mg, 0.844 mmol), HOBt (105 mg, 0.773 mmol) and DIPEA (182 mg, 1.41 mmol) were added. The solution was stirred at room temperature for 4 hours. The solvent was evaporated under reduced pressure and the residue was dissolved in EtOAc (100 mL) and washed with $KHSO_4$ (5%, 3 x 20 mL), $NaHCO_3$ (5%, 3 x 20 mL) and brine (3 x 20 mL) and dried ($MgSO_4$). The crude product was purified by column chromatography (3.5% MeOH in DCM, silica gel) to yield oxidized Ac-Phe(3-F,4-NO_2)-Leu-5-HTP(ox)-Leu-1-methyloxiranyl **6** as a brown powder (81 mg, 15%). ^{1}H NMR (400 MHz, DMSO-d_6) δ 11.40 (s, 1H, indole-NH), 8.72 (s, 1H, 5-HTP-O*H*), 8.34 (m, 1H, N*H*), 8.11 (t, *J* = 8.2 Hz, 1H, arom-*H*),

8.03 (d, J = 7.4 Hz, 1H, arom-H), 7.81 (m, 1H, arom-H), 7.23 (s, 1H, NH) 7.05 (s, 1H, NH), 6.96 (d, J = 7.1 Hz, 1H, arom-H), 6.84 (d, J = 6.3 Hz, 1H, arom-H), 6.65 (m, J = 6.9 Hz, 2H, NH and arom-H), 4.73 (m, 1H, α-CH), 4.61 (m, 1H, α-CH), 4.34 (m, 2H, 2 x α-CH), 3.94 (m, 1H, indole-CH), 3.22 (d, J = 6.4 Hz, 1H, arom-CH), 3.16 (d, J = 5.0 Hz, 1H, epoxide-H), 3.05 (d, J = 2.9 Hz, 1H, arom-CH), 3.00 (d, J = 5.1 Hz, 1H, epoxide-H), 2.87 (m, 1H, arom-CH), 2.33 (m, 1H, arom-CH), 1.90 (s, 3H, CO-CH_3), 1.62 (m, 2H, 2 x Leu-CH), 1.40 (s, 3H, epoxide-CH_3), 1.33 (m, 4H, 2 x Leu-CH), 0.86 (m, 12H, 4 x Leu-CH_3). ^{13}C NMR (101 MHz, DMSO-d$_6$) δ 208.75, 172.15, 171.12, 169.83, 152.47, 152.34, 146.68, 146.25, 145.20, 137.93, 133.16, 125.62, 125.35, 124.68, 121.74, 118.78, 116.61, 115.44, 59.35, 56.90, 53.60, 52.13, 51.19, 50.26, 48.95, 29.48, 24.94, 24.51, 24.25, 23.68, 23.58, 22.85, 22.56, 21.96, 21.45, 16.98. Molecular Formula $C_{37}H_{47}FN_6O_{10}$, HRMS calcd for $[M+H]^+$ 755.3410, found 755.3623 $[M+H]^+$.

2.4 Preparation of leucine epoxyketones 8a and 8b.

Scheme 4S. Synthesis of epoxyketones 8a and 8b.[a]

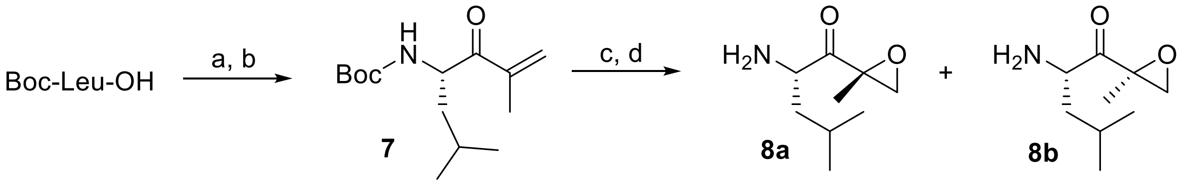

[a]Reagents and conditions: (a) $HN(OCH_3)CH_3$•HCl, EDC, HOBt, DIPEA, DMF, rt, 4 h. (b) isopropenyl MgBr, THF, 0 °C, 6 h. (c) 12.5% NaOCl, pyridine, -5 °C, 2 h. (d) 1:3 TFA/CH_2Cl_2, 0 °C, 2 h.

Boc-Leu-N(CH_3)OCH_3 (not in scheme)**:** Boc-Leu-OH hydrate (10.0 g, 40.1 mmol) was dissolved in DCM (250 mL). BOP (17.7 g, 40.1 mmol), Et_3N (5.59 mL, 40.1 mmol), and *N,O*-dimethylhydroxylamine (3.91 g, 40.1 mmol) were added. Additional Et_3N (~3 mL) was added until the solution reached pH 7. The reaction mixture was stirred at room temperature for 4 hours. The reaction solution was washed with citric acid (10%, 3 x 200 mL), saturated sodium bicarbonate (3 x 200 mL), and brine (3 x 150 mL). The solution was dried ($MgSO_4$) and the solvent was evaporated under reduced pressure. The remaining product was dissolved in ethyl acetate (100 mL) and placed in an ice bath to precipitate residual BOP, which was removed via vacuum filtration. EtOAc was removed under reduced pressure. Precipitation was repeated twice more to

yield a clear, pale yellow syrup (10.2 g, 98.2% yield). ^{1}H NMR (400 MHz, $CDCl_3$) δ 5.06 (d, *J* = 8.8 Hz, 1H, N*H*), 4.75 (s, 1H, α-C*H*), 3.81 (s, 3H, $N(CH_3)$-OCH_3), 3.22 (s, 3H, N(CH_3)-OCH_3), 1.74 (m, 1H, Leu-C*H*), 1.45 (s, 11H, Boc-C(CH_3)$_3$ and Leu-CH_2) 0.99 (d, *J* = 6.5 Hz, 1H, Leu-CH_3), 0.95 (d, *J* = 6.7 Hz, 1H, Leu-CH_3).

Boc-Leu-C(CH_3)=CH_2 (7): Boc-Leu-N(CH_3)OCH_3 (11.7 g, 42.8 mmol) was dissolved in THF (200 mL) in a dry flask at 0 °C. Isopropenyl magnesium bromide (0.5 M in THF, 360 mL, 171 mmol) was added dropwise under an atmosphere of argon. The reaction mixture was stirred below 5 °C for 6 hours, then poured over a slurry of ice (300 mL) and saturated ammonium chloride (300 mL) and stirred for 30 minutes. The THF layer was separated from the solution and the aqueous layer was extracted with EtOAc (3 x 300 mL). The organic layers were combined and washed with water (2 x 200 mL) and brine (2 x 200 mL) and dried ($MgSO_4$). The solvent was evaporated under reduced pressure to yield a green, viscous liquid. The crude product was purified by column chromatography (15:1 hexanes/ethyl acetate, silica gel) to yield a clear, pale yellow liquid (5.37 g, 49.2% yield). ^{1}H NMR (400 MHz, $CDCl_3$) δ 6.10 (s, 1H, C(CH_3)=C*H*), 5.90 (s, 1H, C(CH_3)=C*H*), 5.16 (d, *J* = 8.5 Hz, 1H, NH), 5.08 (m, 1H, α-C*H*), 1.92 (s, 3H, C(CH_3)=CH_2), 1.73 (m, 1H, Leu-C*H*), 1.45 (s, 9H, Boc-C(CH_3)$_3$), 1.33 (m, 2H, Leu-CH_2), 1.02 (d, *J* = 6.5 Hz, 3H, Leu-CH_3), 0.92 (d, *J* = 6.7 Hz, 3H, Leu-CH_3).

Boc-Leu-1-methyloxiranyl *R* and *S* isomers: Boc-Leu-isopropene **7** (5.37 g, 21.1 mmol) was dissolved in pyridine (74 mL) at -5 °C. Aqueous sodium hypochlorite (12.5% by weight, 33.3 mL, 52.6 mmol) was added dropwise to the solution and stirred for two hours while maintaining a reaction mixture temperature below -5 °C. The reaction solution was diluted with EtOAc (100 mL) and washed with water (3 x 100 mL) and brine (2 x 100 mL). The solution was dried ($MgSO_4$) and the solvent was evaporated under reduced pressure to yield a clear orange oil. The crude product was purified by column chromatography (9:1 hexanes/ethyl acetate, silica gel) to yield both isomers as a clear oil in a 2.1 *R* : 1.0 *S* ratio. (1.20 g, 21.1% yield *R-isomer* and 0.58 g, 10.2% *S*-isomer). *R*-isomer: ^{1}H NMR (400 MHz, $CDCl_3$) δ 4.86 (d, *J* = 8.4 Hz, 1H, N*H*), 4.34 (m, 1H, α-C*H*), 3.31 (d, *J* = 4.9 Hz, 1H, epoxide CH_2), 2.91 (d, *J* = 5.0 Hz, 1H, epoxide-CH_2), 1.74 (m, 1H, Leu-C*H*), 1.54 (s, 3H, epoxide-CH_3), 1.43 (s, 9H, Boc-C(CH_3)$_3$), 1.38 (m, 2H, Leu-CH_2), 0.99 (d, *J* = 6.5 Hz, 3H, Leu-CH_3), 0.96 (d, *J* = 6.7 Hz, 3H, Leu-CH_3). *S*-isomer: ^{1}H NMR (400 MHz,

DMSO-d6) δ 4.89 (s, broad, N*H*), 4.59 (m, 1H, α-C*H*), 3.06 (d, *J* = 5.0 Hz, 1H, epoxide-C*H*), 2.88 (d, *J* = 5.0 Hz, 1H, epoxide- C*H*), 1.73 (m, 1H, Leu-C*H*), 1.59 (s, 3H, epoxide-CH_3), 1.46 (s, 9H, Boc-C(CH_3)$_3$), 1.37 (m, 2H, Leu-CH_2), 1.06 (d, *J* = 6.7 Hz, 3H, Leu-CH_3), 0.94 (d, *J* = 6.7 Hz, 3H, Leu-CH_3).

TFA • Leu-1-methyloxiranyl 8a and 8b: Boc-Leu-1-methyloxiranyl (0.670 g, 2.47 mmol) was dissolved in 1:3 TFA/DCM (50 mL) that was pre-cooled to 0 °C. The reaction mixture was stirred for 2 hours and the solvent was evaporated under reduced pressure. The product was washed with DCM (4 x 50 mL) until a white solid formed (510 mg, 72% yield). **8a** ^{1}H NMR (400 MHz, $CDCl_3$) δ 4.06 (dd, *J* = 9.7, 3.2 Hz, 1H, α-C*H*), 3.15 (d, *J* = 4.5 Hz, 1H, epoxide-C*H*), 2.95 (d, *J* = 4.5 Hz, 1H, epoxide-CH_2), 1.86 (m, 1H, Leu-C*H*), 1.71 (m, 1H, Leu-CH_2), 1.57 (m, 1H, 1H, Leu-CH_2 and s, 3H, epoxide-CH_3), 1.01 (d, *J* = 3.2 Hz, 3H, Leu-CH_3), 0.99 (d, *J* = 3.2 Hz, 3H, Leu-CH_3). **8b** ^{1}H NMR (400 MHz, $CDCl_3$) δ 4.86 (d, *J* = 8.5 Hz, 1H, N*H*), 4.22 (m, 1H, α-C*H*), 3.31 (d, *J* = 4.9 Hz, 1H, epoxide-C*H*), 2.91 (d, *J* = 5.0 Hz, 1H, epoxide-C*H*), 1.74 (m, 1H, Leu-C*H*), 1.43 (s, 3H, epoxide-CH_3), 1.21 (m, 2H, Leu-CH_2), 0.99 (d, *J* = 6.5 Hz, 3H, Leu-CH_3), 0.95 (d, *J* = 6.7 Hz, 3H, Leu-CH_3).

2.5 <u>Preparation of macrocyclic peptide amide</u>

Scheme 5S. Synthesis of macrocyclic peptide amide 12.[a]

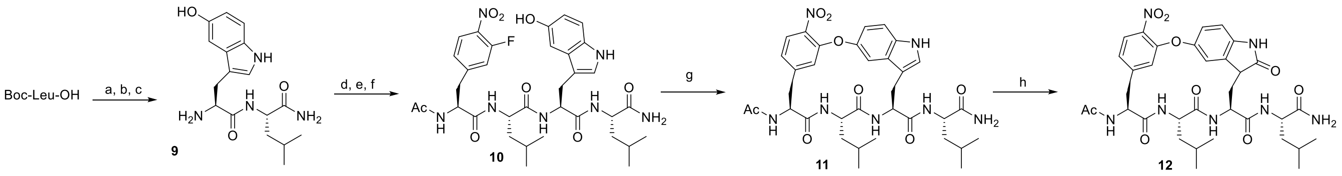

[a]Reagents and conditions: (a) HOBt-NH_3, EDC, DMF, rt, 24 h. (b) (i) 1:3 TFA/CH_2Cl_2, 0 °C, 2 h. (ii) Boc-5-HTP-OH, EDC, HOBt, DIPEA, DMF, rt, 4 h. (c) 1:3 TFA/CH_2Cl_2, 0 °C, 2 h. (d) Boc-Leu-OH, EDC, HOBt, DIPEA, DMF, rt, 4 h. (e) 1:3 TFA/CH_2Cl_2, 0 °C, 2 h. (f) Ac-Phe(3-F,4-NO_2)-OH, EDC, HOBt, DIPEA, DMF, rt, 8 h. (g) molecular sieves (3 Å), K_2CO_3, $CaCO_3$, DMF, 45 °C, 2 wks. (h) DMSO, HCl, Phenol, AcOH, rt, 4 h.

HOBt·NH_3: HOBt (4.71 g, 34.9 mmol) was suspended in H_2O (30 mL) and ammonium hydroxide (29% in H_2O, 3.41 mL, 52.3 mmol) was added. The solution turned clear and was stirred for 10

minutes, concentrated under vacuum, and HOBt-NH_3 was precipitated by addition of acetone and collected by filtration (4.93 g, 93% yield).

Boc-Leu-NH_2 (not shown in scheme): Boc-Leu-OH (5.00 g, 20.1 mmol), HOBt-NH_3 (4.58 g, 30.1 mmol), EDC (4.61 g, 24.1 mmol), and DIPEA (7.0 mL, 40 mmol) were dissolved in DMF (100 mL) and stirred for 12 hours. The solvent was removed under reduced pressure and the solids were dissolved in EtOAc (150 mL), washed with $KHSO_4$ (5%, 3 x 50 mL), $NaHCO_3$ (5%, 3 x 50 mL), brine (2 x 50 mL), and dried over $MgSO_4$. The solvent was removed under reduced pressure to give Boc-Leu-NH_2 (4.46 g, 90% yield) as a white solid. ^{1}H NMR (400 MHz, DMSO-d_6) δ 7.20 (s, 1H, N*H*), 6.91 (s, 1H, N*H*), 6.76 (d, *J* = 8.5 Hz, 1H, N*H*), 3.88 (m, 1H, α-C*H*), 1.58 (m, 1H, Leu-C*H*), 1.38 (m, 11H, Boc-C(CH_3)$_3$ and Leu-CH_2), 0.86 (m, 6H, Leu-CH_3).

Boc-5-HTP-Leu-NH_2 (not shown in scheme): Boc-Leu-NH_2 (2.07 g, 18.2 mmol) was dissolved in TFA:DCM (1:3, 50 mL) and stirred for two hours. The solvent was removed under reduced pressure and the residue co-evaporated with *tert*-butyl methyl ether:hexanes (2:1, 3x10 mL) and dried under vacuum. The resulting solid (4.43 g, 18.2 mmol), Boc-5-HTP-OH (5.82 g, 18.2 mmol), HOBt (2.70 g, 20.0 mmol), EDC (3.10 g, 20.0 mmol), and DIPEA (7.0 mL, 40 mmol) were dissolved in DMF. The pH was maintained at 8-9 by addition of additional DIPEA and the solution stirred for 12 hours. The solvent was removed under reduced pressure and the crude residue dissolved in EtOAc (250 mL), washed with $KHSO_4$ (5%, 3 x 50 mL), $NaHCO_3$ (5%, 3 x 50 mL), brine (3 x 50 mL), and dried over $MgSO_4$. The solvent was removed under reduced pressure to give Boc-5-HTP-Leu-NH_2 (6.86 g, 87% yield) as an off-white solid. ^{1}H NMR (400 MHz, DMSO-d_6) δ 10.49 (s, 1H, indole-N*H*), 8.58 (s, 1H, indole-O*H*), 7.78 (d, *J* = 8.4 Hz, 1H, N*H*), 7.23 (s, 1H, NH_2), 7.11 (d, *J* = 8.6 Hz, arom-*H*), 7.02 (d, *J* = 1.9 Hz, arom-*H*), 7.00 (s, 1H, NH_2), 6.88 (m, 2H, arom-*H* and N*H*) 6.59 (dd, *J* = 6.4, 2.2 Hz, 1H, arom-*H*), 4.27 (m, 1H, α-C*H*), 4.15 (m, 1H, α-C*H*), 2.98 (dd, *J* = 14.6, 4.5 Hz, 1H, arom-CH_2), 2.80 (dd, *J* = 14.6, 9.6 Hz, 1H, arom-CH_2), 1.61 (m, 1H, Leu-C*H*), 1.47 (m, 2H, Leu-CH_2), 1.33 (s, 9H, Boc-C(CH_3)$_3$), 0.88 (d, *J* = 6.5 Hz, 3H, Leu-CH_3), 0.84 (d, *J* = 6.5 Hz, 3H, Leu-CH_3).

Boc-Leu-5-HTP-Leu-NH_2 (not shown in scheme): Boc-5-HTP-Leu-NH_2 (6.86 g, 15.9 mmol) was dissolved in TFA:DCM (1:3, 75 mL) and stirred for two hours. The solvent was removed

under reduced pressure and the residue co-evaporated with *tert*-butyl methyl ether:hexanes (2:1, 3x10 mL) and dried under vacuum. The resulting solid (7.10 g, 15.9 mmol), Boc-Leu-OH (3.95 g, 15.9 mmol), HOBt (2.95 g, 17.5 mmol), EDC (3.35 g, 17.5 mmol), and DIPEA (5.5 mL, 32 mmol) were dissolved in DMF. The pH was maintained at 8-9 by addition of additional DIPEA and the solution stirred for 12 hours. The solvent was removed under reduced pressure and the crude residue dissolved in EtOAc (200 mL), washed with $KHSO_4$ (5%, 3 x 75 mL), $NaHCO_3$ (5%, 3 x 75 mL), brine (3 x 50 mL), and dried over $MgSO_4$. The solvent was removed under reduced pressure to give Boc-Leu-5-HTP-Leu-NH_2 (7.30 g, 84% yield) as an off-white solid. ^{1}H NMR (400 MHz, DMSO-d_6) δ 10.47 (s, 1H, indole-N*H*), 8.54 (s, 1H, indole-O*H*), 7.84 (m, 2H, 2 x N*H*), 7.09 (m, 2H, 2 x N*H*), 7.01 (d, *J* = 2.2 Hz, 1H, arom-*H*), 6.94 (m, 2H, arom-*H*), 6.85 (s, 1H, NH_2), 6.57 (dd, *J* = 6.3, 2.3 Hz, 1H, arom-*H*), 4.53 (m, 1H, α-C*H*), 4.21 (m, 1H, α-C*H*), 3.90 (m, 1H, α-C*H*), 3.04 (dd, *J* = 14.8, 5.6 Hz, 1H, arom-CH_2), 2.85 (dd, *J* = 14.7, 8.2 Hz, 1H, arom-CH_2), 1.52 (m, 2H, 2 x Leu-C*H*), 1.42 (m, 4H, 2 x Leu-CH_2), 1.36 (s, 9H, Boc-C(CH_3)$_3$), 0.83 (m, 12H, 4 x Leu-CH_3).

Ac-Phe(3-F,4-NO_2)-Leu-5-HTP-Leu-NH_2 (10): Boc-Leu-5-HTP-Leu-NH_2 (7.30 g, 13.4 mmol) was dissolved in TFA:DCM (1:3, 75 mL) and stirred for two hours. The solvent was removed under reduced pressure and the residue triturated and co-evaporated with *tert*-butyl methyl ether:hexanes (2:1, 3x15 mL) and dried under vacuum. The residue was then dissolved in EtOAc (10 mL), precipitated by addition of *tert*-butyl methyl ether:hexanes (2:1), and centrifuged. The supernatant was poured off and the solids dried under vacuum, giving crude TFA-Leu-5-HTP-Leu-NH_2 (7.23 g, 99% crude yield). TFA-Leu-5-HTP-Leu-NH_2 (1.54 g, 2.75 mmol), **7S** (0.742 g, 2.75 mmol), HOBt (0.51 g, 3.0 mmol), EDC (0.56 g, 3.0 mmol), and DIPEA (0.96 mL, 5.5 mmol) were dissolved in DMF. The pH was maintained at 8-9 by addition of additional DIPEA and the solution stirred for 12 hours. The solvent was removed under reduced pressure and the crude residue dissolved in EtOAc (75 mL), washed with $KHSO_4$ (5%, 3 x 50 mL), $NaHCO_3$ (5%, 3 x 50 mL), brine (3 x 30 mL), and dried over $MgSO_4$. The solvent was removed under reduced pressure to give tetrapeptide amide **10** (1.54 g, 80% yield) as a tan solid. ^{1}H NMR (400 MHz, DMSO-d_6) δ 10.47 (s, 1H, indole-N*H*), 8.56 (s, 1H, indole-O*H*), 8.37 (s, 1H, N*H*), 8.10 (m, 3H, 2 x N*H*, arom-*H*), 7.83 (d, *J* = 8.2 Hz, 1H, N*H*), 7.46 (d, *J* = 12.2 Hz, 1H, arom-*H*), 7.32 (d, *J* = 8.3 Hz, 1H, arom-*H*), 7.04 (m, 4H, 2 x N*H* and 2 x arom-*H*), 6.85 (d, *J* = 1.9 Hz, 1H, arom-*H*), 6.56 (dd, *J* =

8.6, 2.1 Hz, 1H, arom-*H*), 4.53 (m, 1H, α-C*H*), 4.34 (m, 1H, α-C*H*), 4.22 (m, 2H, 2 x α-C*H*), 3.02 (m, 2H, arom-CH_2), 2.78 (m, 2H, arom-CH_2), 1.89 (s, 3H, CO-CH_3), 1.57 (m, 2H, 2 x Leu-C*H*), 1.43 (m, 4H, 2 x Leu-CH_2), 0.84 (m, 12H, 4 x Leu-CH_3). Molecular formula $C_{34}H_{44}FN_7O_8$, ESI-MS calcd for 697.3, found 698.4 $[M+H]^+$.

Macrocycle Ac-Phe(3-F,4-NO_2)-Leu-5-HTP-Leu-NH_2 (11): Linear tetrapeptide **10** (0.70 g, 1.00 mmol) in DMF (250 mL) was added to molecular sieves (3 Å, 5.0 g), K_2CO_3 (0.38 g, 3.0 mmol), and $CaCO_3$ (0.30 g, 3.0 mmol) previously dried at 170 °C under vacuum for 5 hours, then stirred for 2 weeks at 45 °C. The solution was filtered over Celite and the solvent removed under reduced pressure to give macrocycle **11** as a tan powder (0.54 mg, 79% yield). ^{1}H NMR (400 MHz, DMSO-d_6): δ 11.06 (s, 1H, indole N*H*), 8.35 (d, *J* = 8.9 Hz, 1H, N*H*), 7.93 (m, 3H, 2 x N*H* and arom-*H*), 7.79 (d, *J* = 7.2 Hz, 1H, N*H*), 7.55 (d, *J* = 1.7 Hz, 1H, arom-*H*), 7.37 (m, 2H, N*H* and arom-*H*), 7.30 (d, *J* = 1.7 Hz, 1H, N*H*), 7.01 (s, 1H, arom-*H*), 6.96 (d, *J* = 8.4 Hz, 1H, arom-*H*), 6.75 (dd, *J* = 6.7, 2.0 Hz, 1H, arom-*H*), 6.29 (s, 1H, arom-*H*), 4.82 (m, 1H, α-C*H*), 4.51 (m, 1H, α-C*H*), 4.27 (m, 2H, 2 x α-C*H*), 3.05 (m, 2H, arom-CH_2), 2.87 (dd, *J* = 13.0, 7.2 Hz, 1H, arom-CH_2), 2.66 (dd, *J* = 10.8, 2.1 Hz, 1H, arom-CH_2), 1.87 (s, 3H, CO-CH_3), 1.51 (m, 2H, 2 x Leu-C*H*), 1.42 (m, 4H, 2 x Leu-CH_2), 0.82 (m, 12H, 4 x Leu-CH_3). Molecular formula $C_{34}H_{43}N_7O_8$, ESI-MS calcd for 677.3, found 678.4 $[M+H]^+$.

Macrocycle Ac-Phe(3-F,4-NO_2)-Leu-5-HTP(ox)-Leu-NH_2 (12): Macrocycle **11** (0.42 g, 0.62 mmol) was without further purification dissolved in glacial acetic acid (70 mL) and DMSO (0.12 g, 1.6 mmol), concentrated HCl (0.61 g), and phenol (12 mg, 0.12 mmol) were added. The solution was stirred at room temperature for 20 hrs and monitored by LC-MS (reaction complete after 4 hrs). The solvent was removed under reduced pressure and the resulting crude solid purified by column chromatography (silica gel, 8% MeOH in DCM with 1% formic acid) to yield **12** (2.6 mg, 0.60% yield). ^{1}H NMR (400 MHz, methanol-d_4) δ 8.13 (s, 1H, arom-*H*), 7.99 (d, *J* = 8.4 Hz, 1H, arom-*H*), 7.25 (dd, *J* = 8.5, 1.2 Hz, 1H, arom-*H*), 7.13 (dd, *J* = 8.3, 2.3 Hz, 1H, arom-*H*), 7.04 (s, 1H, arom-*H*), 7.00 (d, *J* = 8.4 Hz, 1H, arom-*H*), 4.81 (dd, *J* = 13.1, 4.3 Hz, 2H, 2 x α-C*H*), 4.30 (m, 2H, 2 x α-C*H*), 3.61 (dd, *J* = 8.6, 4.2 Hz, 1H, oxindole-C*H*), 3.06 (m, 1H, arom-CH_2), 2.93 (dd, *J* = 12.4, 3.8 Hz, 1H, arom-CH_2), 2.68 (m, 1H, arom-CH_2), 2.06 (s, 3H, CO-CH_3), 1.82 (m, 1H, arom-CH_2), 1.61 (m, 2H, 2 x Leu-C*H*), 1.51 (m, 4H, 2 x Leu-CH_2), 0.89 (m, 12H, 4 x Leu-

CH_3). ^{13}C NMR (101 MHz, methanol-d4) δ 175.9, 172.2, 171.4, 170.8, 150.8, 150.2, 143.9, 138.9, 125.7, 122.9, 120.0, 119.4, 116.8, 110.6, 54.1, 52.3, 51.6, 49.5, 49.0, 42.5, 40.7, 31.8, 24.3, 24.1, 22.1, 21.6, 21.2, 20.3. Molecular formula $C_{34}H_{43}N_7O_9$, HRMS calcd for $[M+H]^+$ 694.3195, found 694.3207 $[M+H]^+$.

2.6 Preparation of macrocyclic biphenyl ether epoxyketones 15a and 15b

Scheme 6S: Preparation of biphenyl ether epoxyketone, lacking the oxindole carbonyl.

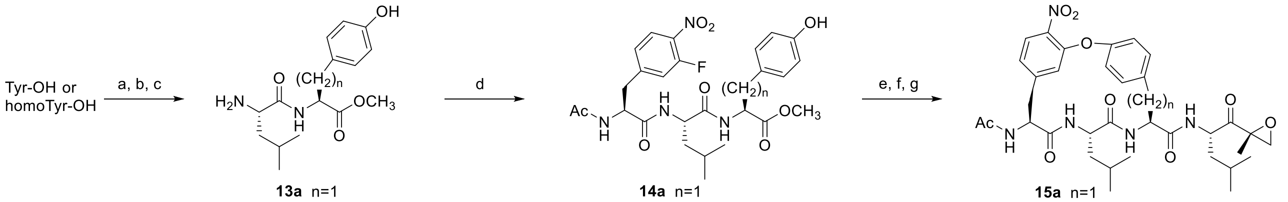

[a]Reagents and conditions: (a) $SOCl_2$, MeOH, -20 °C, 3 h. (b) Boc-Leu-OH, HOBt, DIPEA, EDC, DMF, rt, 4 h. (c) 1:3 TFA/CH_2Cl_2, 0 °C, 2 h. (d) Ac-Phe(3-F,4-NO_2)-OH, EDC, HOBt, DIPEA, DMF, rt, 4 h. (e) molecular sieves (3 Å), K_2CO_3, $CaCO_3$, DMF, 45 °C, 3 days. (f) MeOH, NaOH, rt, 8 h. (g) **8a**, EDC, HOBt, DIPEA, rt, 4 h.

Tyr-OMe (Not numbered in scheme)**:** Tyr-OH (10.00 g, 55.19 mmol) was dissolved in MeOH (250 mL) and cooled to -20 °C. The solution was stirred for 10 minutes, thionyl chloride (12.0 mL, 166 mmol) was added dropwise and the solution was stirred for an additional 3 hours at room temperature. The solvent was evaporated under reduced pressure and the solids were washed with EtOAc to yield Tyr-OMe (17.3 g, 100% yield) as a white solid. ^1H NMR (400 MHz, DMSO-d_6): δ 9.45 (s, 1H, Ph-O*H*), 8.52 (s, 3H, NH_3), 7.01 (d, *J* = 8.5 Hz, 2H, Ph-*H*), 6.72 (d, *J* = 8.5 Hz, 2H, Ph-*H*), 4.19 (t, *J* = 5.1 Hz, 1H, α-C*H*), 3.68 (s, 3H, O-CH_3), 3.02 (m, 2H, Ph-CH_2).

Boc-Leu-Tyr-OMe (Not numbered in scheme)**:** Tyr-OMe (12.8 g, 55.2 mmol) followed by Boc-Leu-OH (13.8 g, 55.2 mmol), EDC • HCl (12.7 g, 66.2 mmol), HOBt (20% hydrate, 10.3 g, 60.7 mmol) and DIPEA (19.2 mL, 110.4 mmol) were dissolved in DMF (250 mL). The pH was adjusted to 8-9 with DIPEA and the solution was stirred overnight. The solvent was removed under reduced pressure and the solids were re-dissolved in EtOAc (300 mL). The solution was washed with $KHSO_4$ (5%, 3 x 100 mL), $NaHCO_3$ (5%, 3 x 100 mL), and brine (3 x 100 mL) and dried with anhydrous $MgSO_4$. The solvent was removed under reduced pressure yielding Boc-Leu-Tyr-OMe

(18.7 g, 85% yield) as a dark orange syrup. ^{1}H NMR (400 MHz, DMSO-d_6) δ 9.23 (s, 1H, Tyr-O*H*), 8.03 (d, *J* = 7.6 Hz, 1H, N*H*), 6.98 (d, *J* = 8.4 Hz, 2H, arom-*H*), 6.83 (d, *J* = 8.6 Hz, 1H, N*H*), 6.64 (d, *J* = 8.5 Hz, 2H, arom-*H*), 4.39 (q, *J* = 7.1 Hz, 1H, α-C*H*), 3.96 (q, *J* = 7.9 Hz, 1H, α-C*H*), 3.57 (s, 3H, OCH_3), 2.84 (m, 2H, Tyr-CH_2), 1.53 (m, 1H, Leu-C*H*), 1.37 (s, 9H, Boc-C$(CH_3)_3$), 1.32 (m, 2H, Leu-CH_2), 0.85 (2d, *J* = 6.5 Hz, 6H, 2 x Leu-CH_3).

TFA • Leu-Tyr-OMe (13a): Boc-Leu-Tyr-OMe (18.7 g, 45.7 mmol) was dissolved in a cooled DCM/TFA solution (3:1, 150 mL) and stirred overnight. The solvent was removed under reduced pressure yielding the dark orange oil Leu-Tyr-OMe **13a** (9.20 g, 48%). ^{1}H NMR (400 MHz, DMSO-d_6) δ 8.88 (d, *J* = 7.6 Hz, 1H, N*H*), 8.10 (s, 3H, NH_3), 7.02 (d, *J* = 8.4 Hz, 2H, arom-*H*), 6.66 (d, *J* = 8.5 Hz, 2H, arom-*H*), 4.45 (q, *J* = 7.1 Hz, 1H, α-C*H*), 3.78 (m, 1H, α-C*H*), 3.59 (s, 3H, OCH_3), 2.84 (m, 2H, Tyr-CH_2), 1.64 (m, 1H, Leu-C*H*), 1.56 (m, 2H, Leu-CH_2), 0.85 (2d, *J* = 6.8 Hz, 6H, 2 x Leu-CH_3).

Ac-Phe(3-F,4-NO_2)-Leu-Tyr-OMe (14a): TFA • Leu-Tyr-OMe **13a** (9.20 g, 21.8 mmol) followed by (S)-*N*-acetyl-3-fluoro-4-nitrophenylalanine **7S** (5.88 g, 21.8 mmol), EDC • HCl (5.01 g, 26.1 mmol), HOBt (20% hydrate, 4.05 g, 24.0 mmol) and DIPEA (5.63 g, 43.6 mmol) were dissolved in DMF. The pH was adjusted to 8-9 with DIPEA and the solution was stirred overnight. The solvent was removed under reduced pressure and the solids were re-dissolved in EtOAc (150 mL). The solution was washed with $KHSO_4$ (5%, 3 x 100 mL), $NaHCO_3$ (5%, 3 x 100 mL) and brine (3 x 100 mL) and dried ($MgSO_4$). The solvent was evaporated under reduced pressure yielding an amber oil. Purification by column chromatography (3.5% MeOH in DCM, silica gel) yielded Ac-Phe(3-F,4-NO_2)-Leu-Tyr-OMe **14a** (4.95 g, 41% yield) as an orange powder. ^{1}H NMR (400 MHz, DMSO-d_6) δ 9.22 (s, 1H, Tyr-O*H*), 8.31 (d, *J* = 8.2 Hz, N*H*), 8.29 (d, *J* = 3.9 Hz, 1H, N*H*), 8.27 (d, *J* = 2.3 Hz, 1H, arom-*H*), 7.44 (t, *J* = 11.5 Hz, 1H, arom-*H*), 7.29 (t, *J* = 9.1 Hz, 1H, arom-*H*), 7.00 (d, *J* = 8.4 Hz, 2H, arom-*H*), 6.65 (d, *J* = 8.4 Hz, 2H, arom-*H*), 4.62 (m, 1H, α-C*H*), 4.38 (m, 1H, α-C*H*), 4.31 (m, 1H, α-C*H*), 3.54 (s, 3H, OCH_3), 2.88-3.04 (m, 4H, 2 x arom-CH_2), 1.80 (s, 3H, CO-CH_3), 1.56 (m, 1H, Leu-C*H*), 1.30 (m, 2H, Leu-CH_2), 0.87 (2d, *J* = 6.5 Hz, 6H, 2 x Leu-CH_3).

Macrocyclized Ac-Phe(3-F,4-NO_2)-Leu-Tyr-OMe (Not numbered in schemes)**:** Molecular sieves (3Å, 12 g), K_2CO_3 (3.66 g, 26.5 mmol) and $CaCO_3$ (2.65 g, 26.5 mmol) were combined and dried by heating at 170 °C under vacuum, in an oil bath for 5 hours. Ac-Phe(3-F,4-NO_2)-Leu-Tyr-OMe **14a** (4.95 g, 8.82 mmol) in DMF (300 mL) was added and the solution was kept at 45 °C in an oil bath for 3.5 days. Reaction progress was monitored with LC-MS. The reaction mixture was filtered over a zeolite bed and the solvent was removed under reduced pressure. The resulting bright orange oil was purified using column chromatography (3.5% MeOH in DCM, silica gel) yielding macrocyclized Ac-Phe(3-F,4-NO_2)-Leu-Tyr-OMe (845 mg, 18% yield) as a pale yellow solid. ^{1}H NMR (400 MHz, DMSO-d_6) δ 8.34 (d, *J* = 10.0 Hz, 1H, N*H*), 8.10 (d, *J* = 9.3 Hz, 1H, N*H*), 7.89 (d, *J* = 8.3 Hz, 1H, N*H*), 7.43 (d, *J* = 8.4 Hz, 1H, arom-*H*), 7.35 (d, *J* = 7.6 Hz, 2H, arom-*H*), 7.11 (d, *J* = 8.3 Hz, 1H, arom-*H*), 6.81 (d, *J* = 8.3 Hz, 1H, arom-*H*), 6.81 (d, *J* = 8.1 Hz, 1H, arom-*H*), 6.70 (d, *J* = 8.5 Hz, 1H, arom-*H*), 4.65 (m, 2H, 2 x α-C*H*), 4.36 (m, 1H, α-C*H*), 3.51 (s, 3H, O-CH_3), 3.13 (dd, *J* = 13.4, 7.5 Hz, 2H, arom-CH_2), 2.67 (m, 2H, arom-CH_2), 1.85 (s, 3H, CO-CH_3), 1.51 (m, 1H, Leu C*H*), 1.41 (m, 2H, Leu-CH_2), 0.87 (d, *J* = 3.5 Hz, 3H, Leu-CH_3), 0.85 (d, *J* = 3.5 Hz, 3H, Leu-CH_3). Molecular formula $C_{27}H_{32}N_4O_8$, ESI-MS calcd for 540.2, found 541.2 $[M+H]^+$.

Macrocyclized Ac-Phe(3-F,4-NO_2)-Leu-Tyr-Leu-1-methyloxiranyl (15a): Macrocyclized Ac-Phe(3-F,4-NO_2)-Leu-Tyr-OMe (845 mg, 1.56 mmol) was suspended in MeOH. NaOH (1 M, 4.69 mL, 4.69 mmol) was added and the mixture was stirred at room temperature overnight. The solvent was removed under reduced pressure and the orange solids were dissolved in H_2O. The solution was acidified to pH 2 with HCl (3 M) then extracted with EtOAc (3 x 75 mL). The combined organic layers were washed with brine (3 x 50 mL), dried ($MgSO_4$), and the solvent was removed under reduced pressure yielding macrocyclized Ac-Phe(3-F,4-NO_2)-Leu-Tyr-OH (650 mg, 79% yield) as a crude yellow powder. Crude macrocyclized Ac-Phe(3-F,4-NO_2)-Leu-Tyr-OH (362 mg, 0.688 mmol) was dissolved in DMF (100 mL) followed by TFA • Leu-1-methyloxiranyl **8a (**196 mg, 0.687 mmol), EDC • HCl (158 mg, 0.824 mmol), HOBt (20% hydrate, 128 mg, 0.756 mmol) and DIPEA (178 mg, 1.37 mmol). The pH was adjusted to 8-9 with DIPEA and the solution was stirred overnight. The solvent was removed under reduced pressure and the solids were re-dissolved in EtOAc (100 mL). The solution was washed with $KHSO_4$ (5%, 3 x 50 mL), $NaHCO_3$ (5%, 3 x 50 mL) and brine (3 x 50 mL) and dried with anhydrous $MgSO_4$. The solvent was

evaporated under reduced pressure yielding a pale yellow solid. The product was precipitated from 3.5% MeOH/DCM to give Ac-Phe(3-F,4-NO_2)-Leu-Tyr-Leu-1-methyloxiranyl **15a** (186 mg, 41% yield) as an off-white powder. ^{1}H NMR (400 MHz, $CDCl_3$) δ 7.96 (s, 1H, N*H*), 7.88 (d, *J* = 8.2 Hz, 2H, arom-*H* and NH), 7.45 (d, *J* = 7.8 Hz, 2H, arom-*H* and N*H*), 7.18 (d, *J* = 7.8 Hz, 1H, N*H*), 6.86 (d, *J* = 6.0 Hz, 2H, arom-*H*), 6.69 (d, *J* = 8.3 Hz, 1H, arom-*H*), 6.34 (d, *J* = 7.0 Hz, 1H, arom-*H*), 5.25 (t, *J* = 6.3 Hz, 1H, α-C*H*), 4.99 (t, *J* = 9.6 Hz, 1H, α-C*H*), 4.52 (m, 2H, 2 x α-C*H*), 3.45 (m, 1H, arom-CH_2), 3.42 (d, *J* = 4.0 Hz, 1H, epoxide-*H*), 2.99 (m, 1H, arom-CH_2), 2.89 (d, *J* = 4.0 Hz, 1H, epoxide-*H*), 2.83 (t, *J* = 12.8 Hz, 1H, arom-CH_2), 2.37 (d, *J* = 13.3 Hz, 2H, arom-CH_2), 1.88 (s, 3H, CO-CH_3), 1.62 (m, 1H, Leu-C*H*), 1.61 (s, 3H, epoxide-CH_3), 1.56 (m, 1H, Leu-C*H*), 1.24-1.40 (m, 4H, 2 x Leu-CH_2), 0.87 (d, *J* = 6.5 Hz, 3H, Leu-CH_3), 0.84 (d, *J* = 6.6 Hz, 3H, Leu-CH_3), 0.81 (d, *J* = 4.6 Hz, 3H, Leu-CH_3), 0.73 (d, *J* = 4.9 Hz, 3H, Leu-CH_3). ^{13}C NMR (101 MHz, $CDCl_3$) δ 207.36, 172.66, 170.21, 168.53, 153.29, 152.70, 142.63, 138.14, 135.01, 132.48, 130.58, 128.55, 125.66, 122.83, 122.35, 121.73, 117.59, 108.19, 59.44, 55.43, 52.95, 51.74, 50.12, 43.96, 39.26, 38.74, 26.02, 24.73, 23.50, 23.42, 22.93, 22.06, 21.41, 17.02. Molecular formula $C_{35}H_{45}N_5O_9$, HRMS calcd for $[M+H]^+$ 680.3290, found 680.3303 $[M+H]^+$.

homoTyr-OMe (Not numbered in scheme)**:** homoTyr-OH•HBr (5.00 g, 18.1 mmol) was dissolved in MeOH (150 mL) and cooled to -20 °C. Thionyl chloride (3.9 mL, 54 mmol) was added dropwise and the solution was stirred for an additional 3 hours at room temperature. The solvent was evaporated under reduced pressure and the solids were washed with EtOAc to yield homoTyr-OMe (6.33 g, 100% yield) as a white solid. ^{1}H NMR (400 MHz, DMSO-d_6): δ 9.26 (s, 1H, Ph-O*H*), 8.52 (s, 3H, NH_3), 7.01 (d, *J* = 8.4 Hz, 2H, Ph-H), 6.70 (d, *J* = 8.4 Hz, 2H, Ph-H), 4.03 (q, *J* = 7.1 Hz, 1H, α-C*H*), 3.76 (s, 3H, O-CH_3), 2.62 (m, 2H, Ph-CH_2), 2.02 (m, 2H, Ph-CH_2CH_2).

Boc-Leu-homoTyr-OMe (Not numbered in scheme)**:** homoTyr-OMe (5.25 g, 18.1 mmol) followed by Boc-Leu-OH (4.53 g, 18.1 mmol), EDC • HCl (4.16 g, 21.7 mmol), HOBt (4.49 g, 19.9 mmol) and DIPEA (4.68 g, 36.2 mmol) were dissolved in DMF (150 mL). The pH was adjusted to 8-9 with DIPEA and the solution was stirred overnight. Solvent was removed under reduced pressure and the solids were re-dissolved in EtOAc (200 mL). The solution was washed with $KHSO_4$ (5%, 3 x 100 mL), $NaHCO_3$ (5%, 3 x 100 mL) and brine (3 x 100 mL) and dried ($MgSO_4$). The solvent was removed under reduced pressure yielding Boc-Leu-homoTyr-OMe

(5.35 g, 70% yield) as a yellow solid. ^{1}H NMR (400 MHz, $CDCl_3$) δ 6.97 (d, *J* = 8.2 Hz, 2H, arom-*H*), 6.76 (d, *J* = 8.2 Hz, 2H, arom-*H*), 5.03 (d, *J* = 5.1 Hz, 1H, α-C*H*), 4.60 (q, *J* = 5.1 Hz, 1H, α-C*H*), 3.73 (s, 3H, OCH_3), 2.56 (t, *J* = 7.7 Hz, 2H, Ph-CH_2), 2.12 (m, 2H, Ph-CH_2CH_2), 1.93 (m, 1H, Leu-C*H*), 1.64 (m, 2H, Leu-CH_2), 1.45 (s, 9H, Boc-C(CH_3)$_3$), 0.95 (2d, *J* = 6.6 Hz, 6H, 2 x Leu-CH_3).

TFA • Leu-homoTyr-OMe (13b): Boc-Leu-homoTyr-OMe (5.35 g, 12.6 mmol) was dissolved in a cooled DCM/TFA solution (3:1, 75 mL) and stirred for 4h. The solvent was removed under reduced pressure and the crude product was triturated with TBME/hexane (2:1) to give Leu-homoTyr-OMe **13b** as a yellow solid (5.53 g, 100%). ^{1}H NMR (400 MHz, DMSO-d_6) δ 8.93 (d, *J* = 7.4 Hz, 1H, N*H*), 8.21 (s, 3H, NH_3), 7.00 (d, *J* = 8.5 Hz, 2H, arom-*H*), 6.68 (d, *J* = 8.5 Hz, 2H, arom-*H*), 4.26 (m, 1H, α-C*H*), 3.78 (m, 1H, α-C*H*), 3.62 (s, 3H, OCH_3), 2.54 (m, 2H, Ph-CH_2), 1.95 (m, 2H, Ph-CH_2CH_2), 1.70 (m, 1H, Leu-C*H*), 1.57 (m, 2H, Leu-CH_2), 0.93 (2d, *J* = 6.5 Hz, 6H, 2 x Leu-CH_3).

Ac-Phe(3-F,4-NO_2)-Leu-homoTyr-OMe (14b): TFA • Leu-homoTyr-OMe **13b** (3.80 g, 8.70 mmol) followed by (S)-*N*-acetyl-3-fluoro-4-nitrophenylalanine **7S** (2.35 g, 8.70 mmol), EDC • HCl (2.00 g, 10.43 mmol), HOBt (1.29 g, 9.57.0 mmol) and DIPEA (1.29 g, 17.4 mmol) were dissolved in DMF (100 mL). The solvent was removed under reduced pressure and the solids were re-dissolved in EtOAc (150 mL). The solution was washed with $KHSO_4$ (5%, 3 x 100 mL), $NaHCO_3$ (5%, 3 x 100 mL) and brine (3 x 100 mL) and dried ($MgSO_4$). The solvent was evaporated under reduced pressure yielding an amber oil. Purification by column chromatography (3.5% MeOH in DCM, silica gel) yielded Ac-Phe(3-F,4-NO_2)-Leu-homoTyr-OMe **14b** (1.26 g, 25% yield). ^{1}H NMR (400 MHz, DMSO-d_6) δ 9.16 (s, 1H, Tyr-O*H*), 8.20 (d, *J* = 7.5 Hz, N*H*), 8.39 (d, *J* = 8.0 Hz, N*H*), 8.16 (d, *J* = 8.4 Hz, 1H, N*H*), 8.08 (t, *J* = 8.3 Hz, 1H, arom-*H*), 7.46 (dd, *J* = 12.6, 1.5 Hz, 1H, arom-*H*), 7.31 (d, *J* = 1.4 Hz, 1H, arom-*H*), 6.96 (d, *J* = 8.4 Hz, 2H, arom-*H*), 6.65 (d, *J* = 8.2 Hz, 2H, arom-*H*), 4.63 (m, 1H, α-C*H*), 4.36 (m, 1H, α-C*H*), 4.17 (m, 1H, α-C*H*), 3.59 (s, 3H, OCH_3), 3.10 (dd, *J* = 13.6, 4.3 Hz, 1H, Ph-C*H*), 2.85 (dd, *J* = 13.6, 9.9 Hz, 1H, Ph-C*H*), 2.45 (m, 2H, Tyr-CH_2), 1.89 (m, 2H, Tyr-CH_2CH_2), 1.76 (s, 3H, CO-CH_3), 1.59 (m, 1H, Leu-C*H*), 1.48 (m, 2H, Leu-CH_2), 0.92 (d, *J* = 6.5 Hz, 3H, Leu-CH_3), 0.88 (d, *J* = 6.5 Hz, 3H, Leu-CH_3).

Macrocyclized Ac-Phe(3-F,4-NO_2)-Leu-homoTyr-OMe (Not numbered in schemes)**:** Molecular sieves (4Å, 5 g), K_2CO_3 (0.90 g, 6.5 mmol) and $CaCO_3$ (0.65 g, 6.5 mmol) were combined and dried by heating at 170 °C under vacuum, in an oil bath for 5 hours. Ac-Phe(3-F,4-NO_2)-Leu-homoTyr-OMe **14b** (1.25 g, 2.18 mmol) and DMF (100 mL) were added and the solution was kept at 45 °C in an oil bath for 3 days. Reaction progress was monitored with LC-MS. The reaction mixture was filtered over a zeolite bed and the solvent was removed under reduced pressure. The resulting bright orange oil was purified using column chromatography (3.5% MeOH in DCM, silica gel) yielding macrocyclized Ac-Phe(3-F,4-NO_2)-Leu-homoTyr-OMe (130 mg, 13% yield) as a yellow solid. Molecular formula $C_{28}H_{34}N_4O_8$, ESI-MS calcd for 554.2, found 555.3 $[M+H]^+$.

Macrocyclized Ac-Phe(3-F,4-NO_2)-Leu-homoTyr-Leu-1-methyloxiranyl (15b): Macrocyclized Ac-Phe(3-F,4-NO_2)-Leu-homoTyr-OMe (130 mg, 0.234 mmol) was suspended in MeOH (20 mL). NaOH (1 M, 1 mL) was added and the mixture was stirred at room temperature overnight. The solvent was removed under reduced pressure and the orange solids were dissolved in H_2O. The solution was acidified to pH 2 with HCl (3 M) then extracted with EtOAc (3 x 75 mL). The combined organic layers were washed with brine (3 x 50 mL), dried over anhydrous $MgSO_4$, and the solvent was removed under reduced pressure yielding macrocyclized Ac-Phe(3-F,4-NO_2)-Leu-homoTyr-OH (100 mg, 79% yield) as a crude yellow powder. Crude macrocyclized Ac-Phe(3-F,4-NO_2)-Leu-homoTyr-OH (100 mg, 0.185 mmol) was dissolved in DMF (50 mL) followed by TFA • Leu-1-methyloxiranyl **8a (**53 mg, 0.185 mmol), EDC • HCl (43 mg, 0.22 mmol), HOBt (28 mg, 0.20 mmol) and DIPEA (48 mg, 0.37 mmol). The pH was adjusted to 8-9 with DIPEA and the solution was stirred overnight. The solvent was removed under reduced pressure and the solids were re-dissolved in EtOAc (50 mL). The solution was washed with $KHSO_4$ (5%, 3 x 30 mL), $NaHCO_3$ (5%, 3 x 30 mL) and brine (3 x 30 mL) and dried with anhydrous $MgSO_4$. The solvent was evaporated under reduced pressure yielding a pale yellow solid. The product was purified by column chromatography (3.5% MeOH/DCM, silica) to give Ac-Phe(3-F,4-NO_2)-Leu-homoTyr-Leu-1-methyloxiranyl **15b** (186 mg, 41% yield) as an off-white powder. ^1H NMR (400 MHz, DMSO-d_6) δ 8.16 (d, J = 8.0 Hz, 1H, N*H*), 7.98 (d, J = 7.5 Hz, 1H, N*H*), 7.94 (d, J = 8.3 Hz, 1H, arom-*H*), 7.88 (d, J = 8.1 Hz, 1H, N*H*), 7.31 (d, J = 9.8 Hz, 1H, N*H*), 7.25 (d, J = 8.4 Hz, 2H, arom-*H*), 7.16 (dd, J = 1.3, 8.4 Hz, 1H, arom-*H*), 6.94 (d, J = 8.2 Hz, 2H, arom-

H), 6.51 (d, *J* = 5.1 Hz, 1H, arom-*H*), 4.36 (m, 1H, α-C*H*), 4.13 (m, 1H, α-C*H*), 4.00 (m, 2H, 2 x α-C*H*), 3.15 (d, *J* = 5.2 Hz, 1H, epoxide-*H*), 3.02 (d, *J* = 5.1 Hz, 1H epoxide-*H*), 2.98 (m, 1H, arom-C*H*), 2.82 (m, 3H, arom-C*H*), 2.15 (m, 1H, Tyr-CH_2C*H*), 1.94 (m, 1H, Tyr-CH_2C*H*), 1.83 (s, 3H, CO-CH_3), 1.65 (m, 1H, Leu-C*H*), 1.41 (s, 3H, epoxide-CH_3), 1.56 (m, 1H, Leu-C*H*), 1.24-1.40 (m, 4H, 2 x Leu-CH_2), 0.90 (d, *J* = 6.6 Hz, 3H, Leu-CH_3), 0.83 (d, *J* = 6.5 Hz, 3H, Leu-CH_3), 0.79 (d, *J* = 6.5 Hz, 6H, Leu-CH_3). ^{13}C NMR (101 MHz, DMSO-d_6) δ 208.89, 173.06, 171.47, 169.88, 169.14, 152.78, 150.31, 145.33, 139.40, 136.35, 131.04, 125.83, 124.45, 120.13, 119.45, 59.34, 56.99, 52.07, 50.99, 49.89, 41.54, 38.93, 30.99, 29.35, 24.98, 24.57, 23.91, 23.67, 23.03, 22.04, 21.35, 16.95. Molecular formula $C_{36}H_{47}N_5O_9$, HRMS calcd for $[M+H]^+$ 694.3447, found 694.3442 $[M+H]^+$.

3. ^{1}H-NMR and ^{13}C-NMR spectra of 5, 6, 12, 15a and 15b

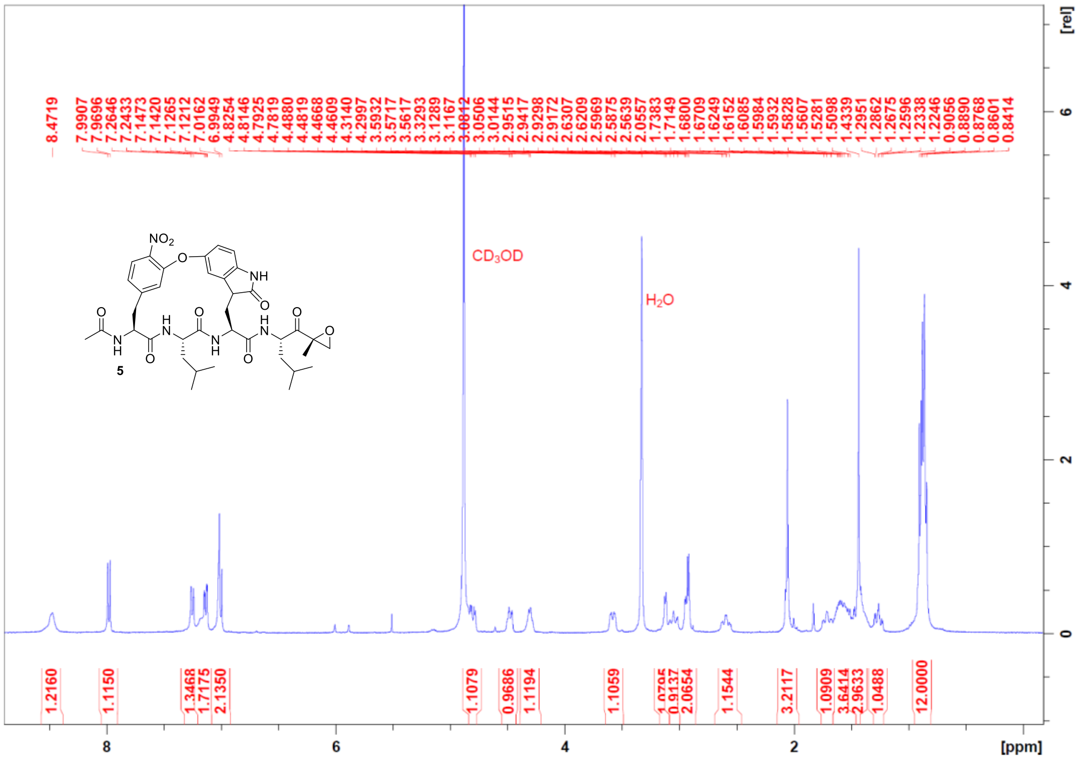

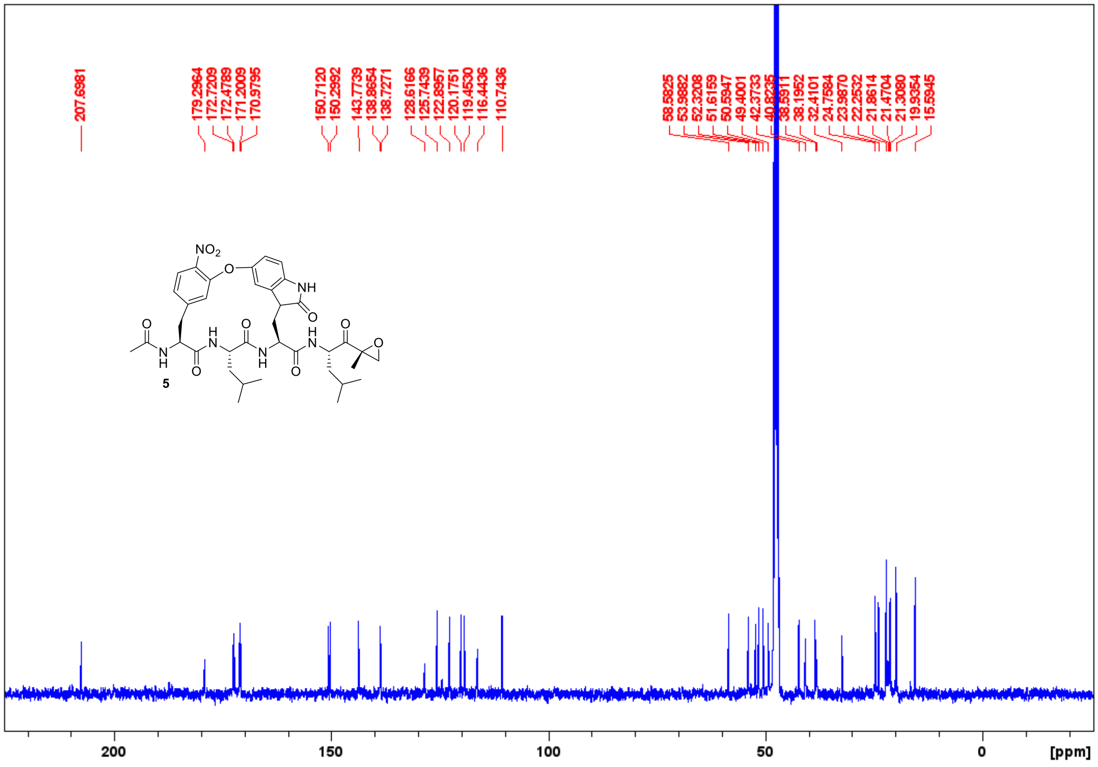

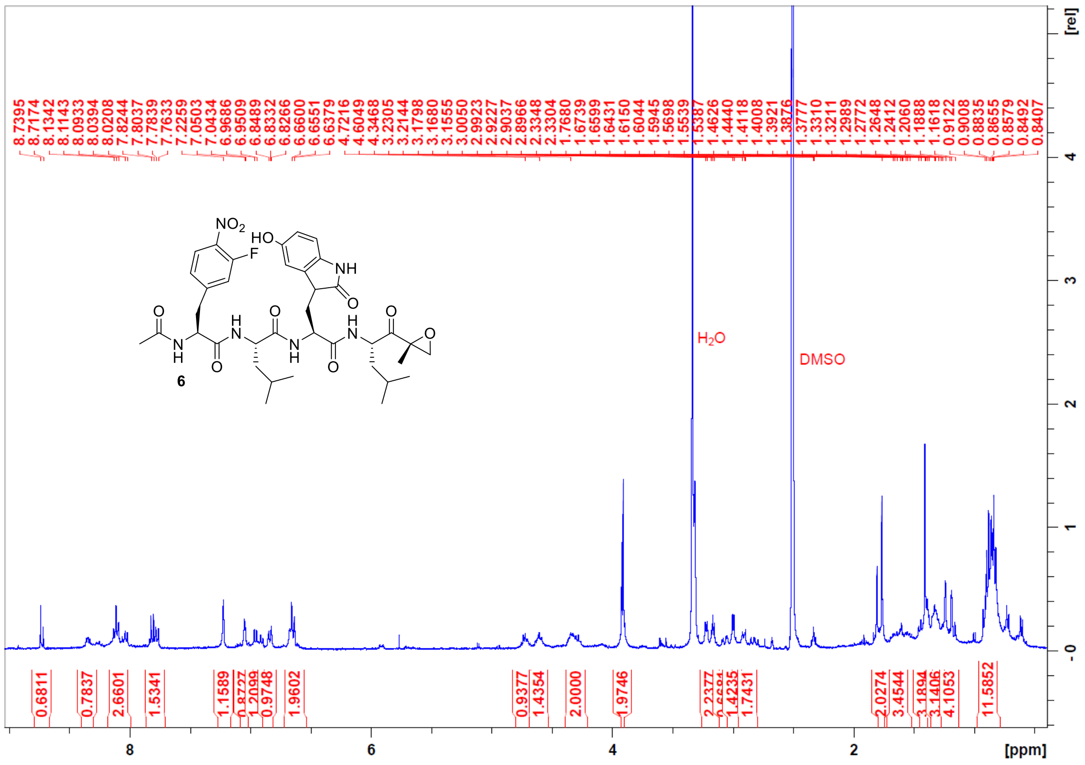
6
H2O
DMSO
[rel]
[ppm]

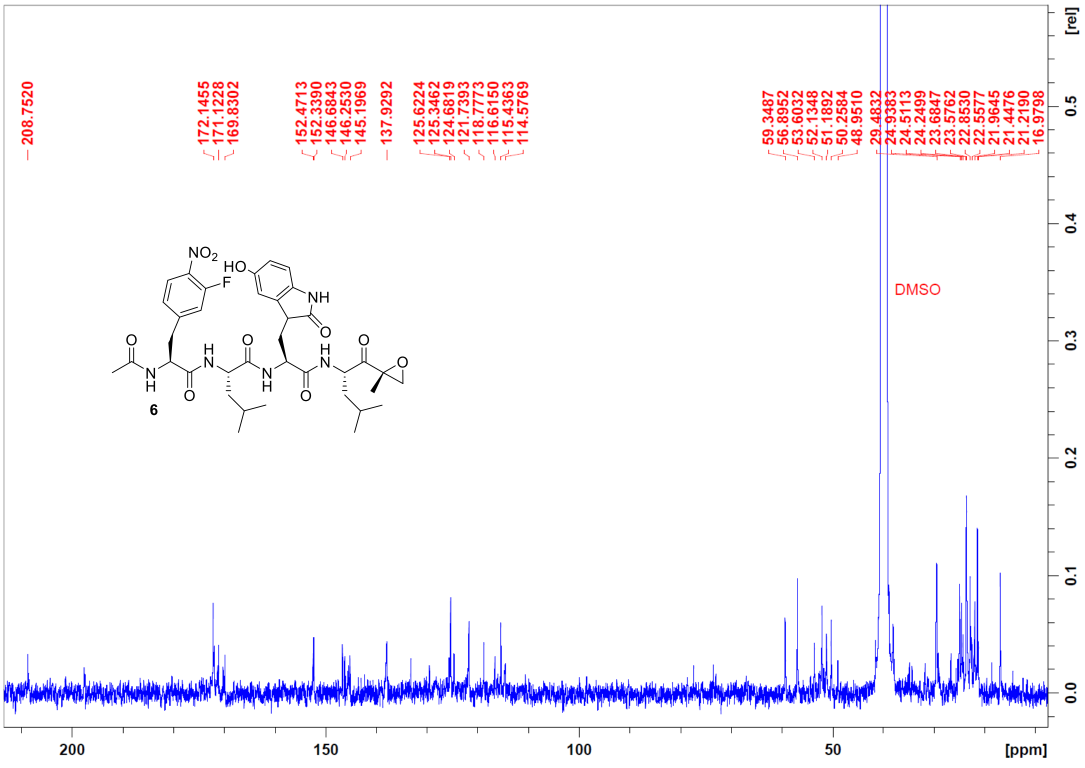
6
DMSO
[rel]
[ppm]

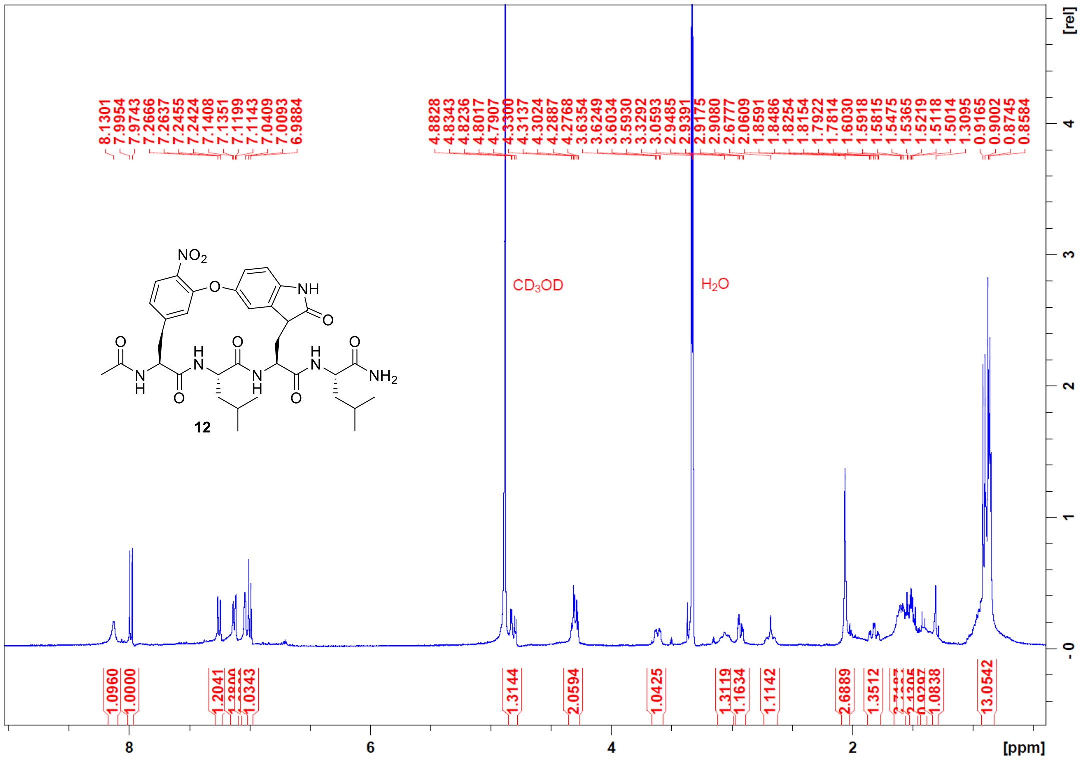
NO2
NH
O
12
CD3OD
H2O
[rel]
[ppm]

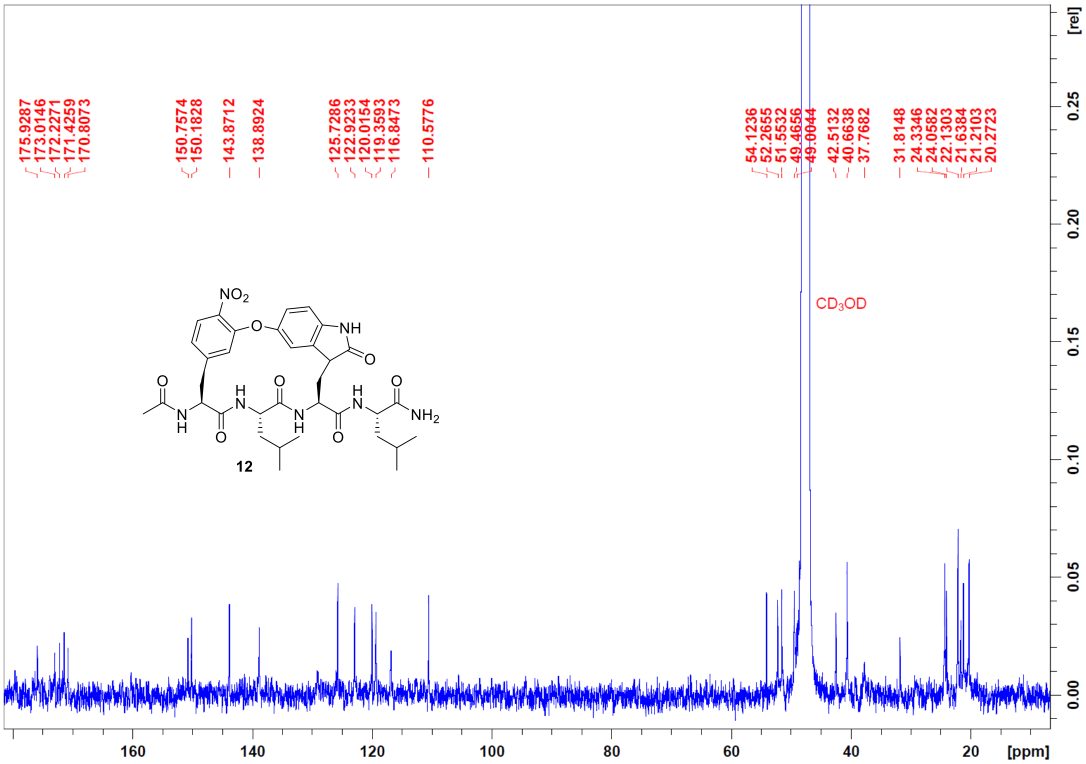
NO2
NH
O
12
CD3OD
[rel]
[ppm]

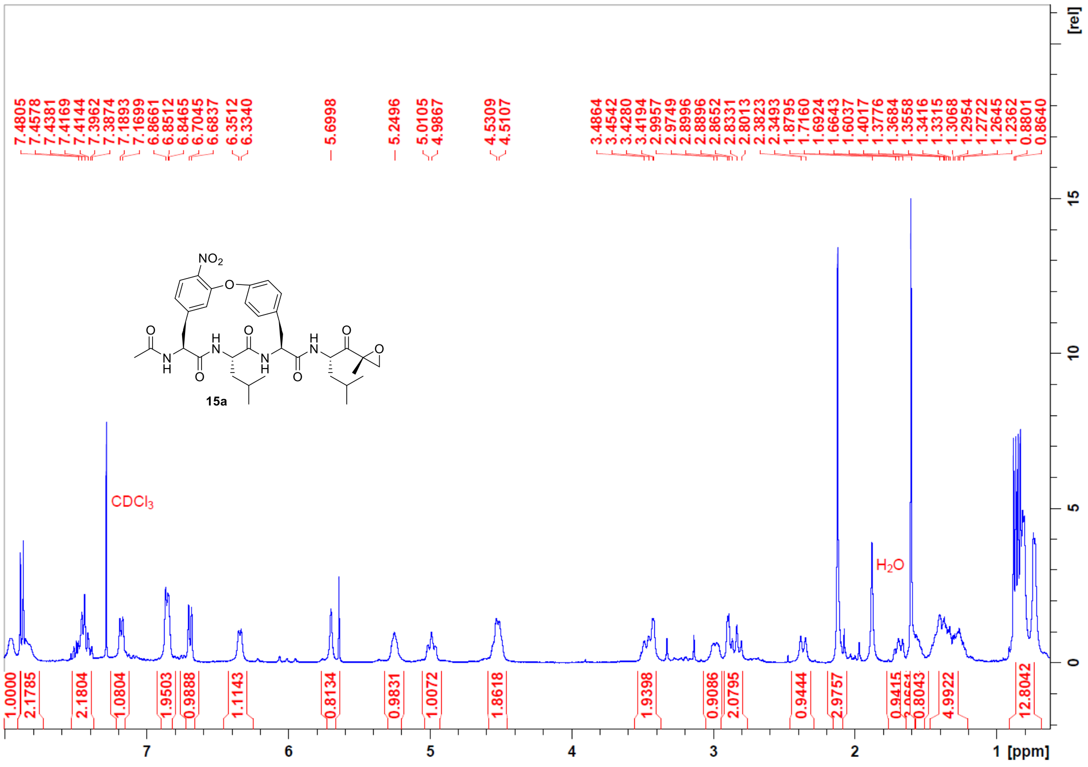
15a
CDCl3
H2O
[rel]
[ppm]

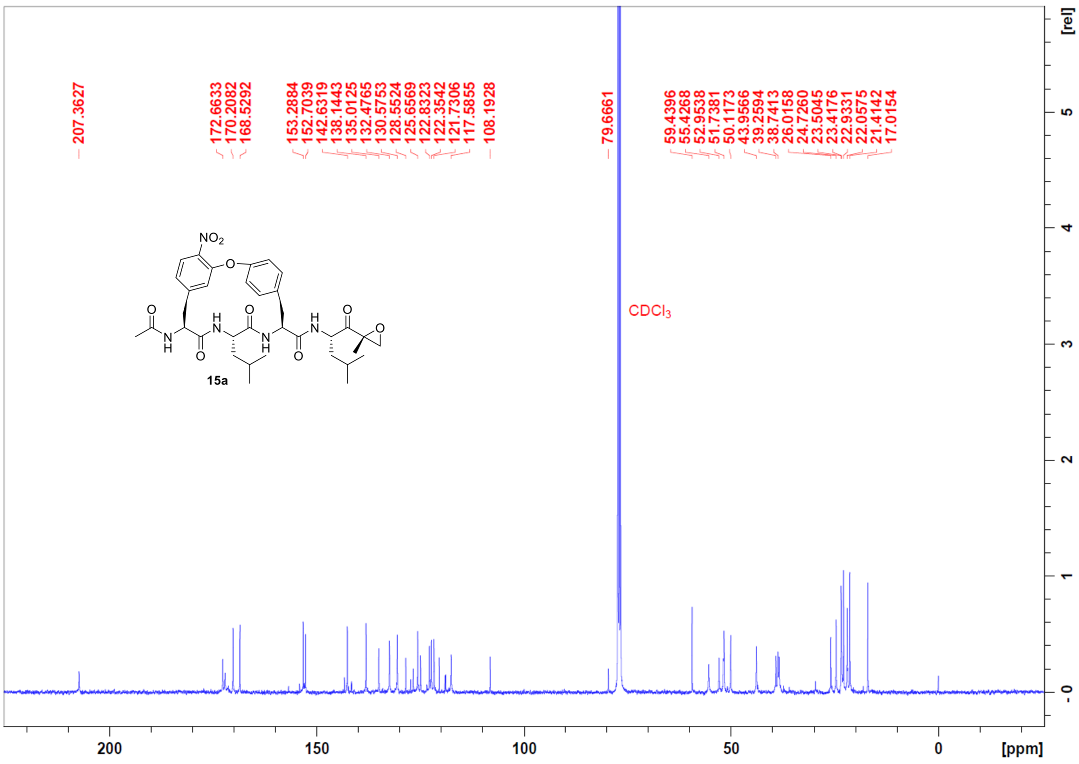
15a
CDCl3
[rel]
[ppm]

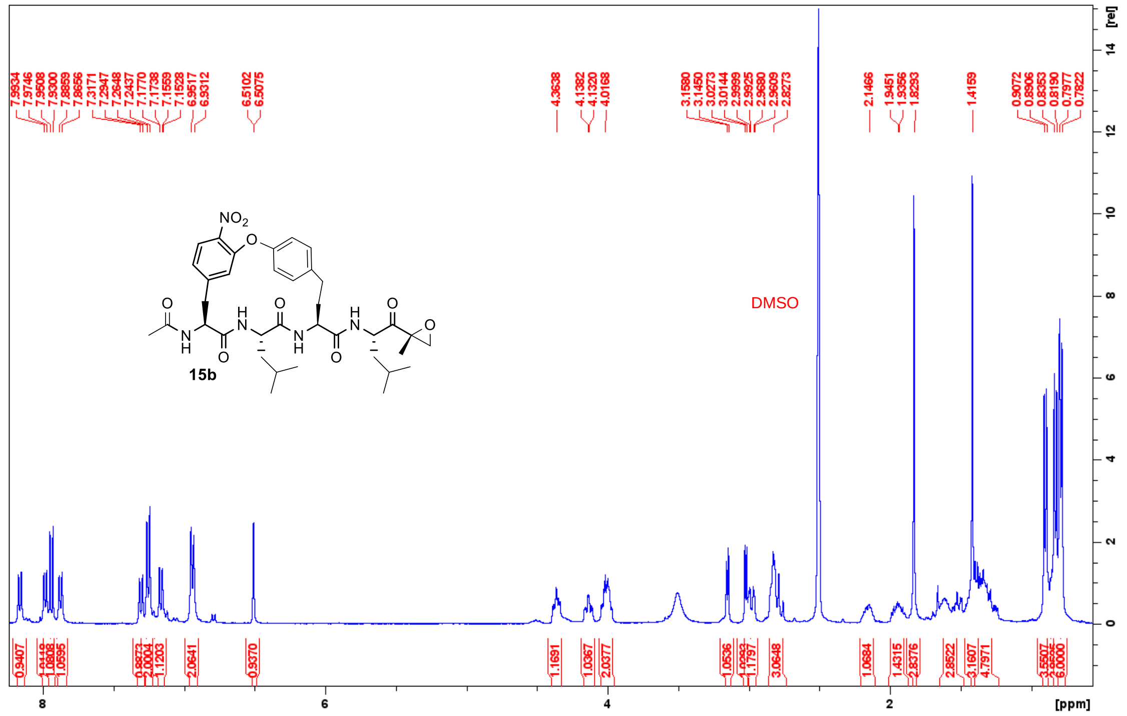

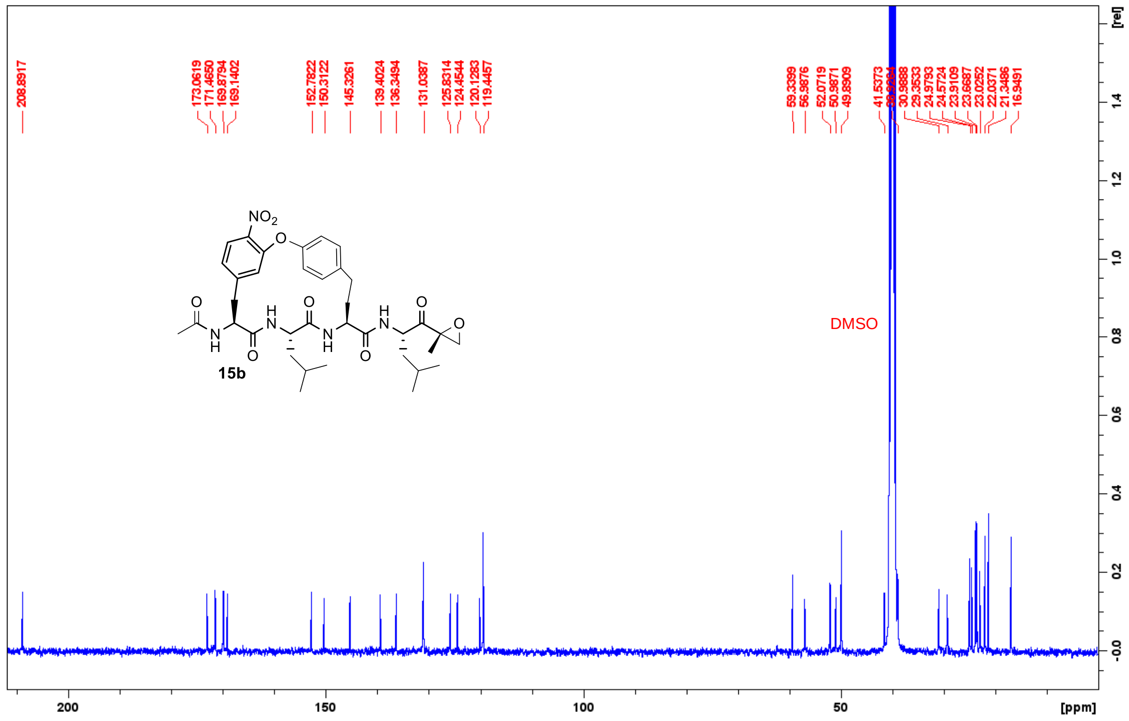

4. HRMS Analysis of Final Compounds

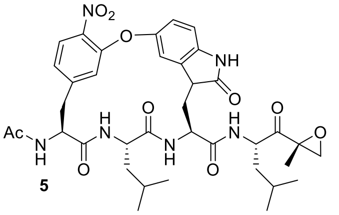

5

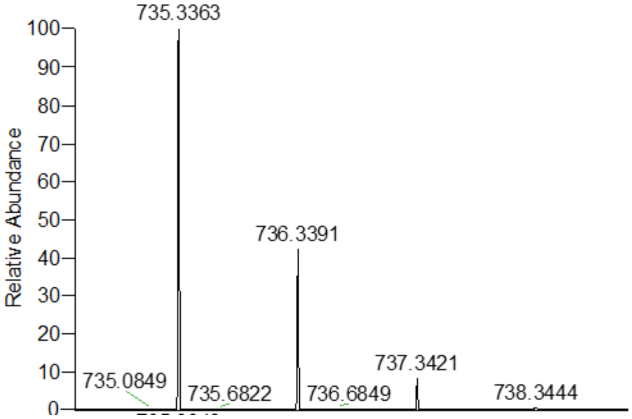

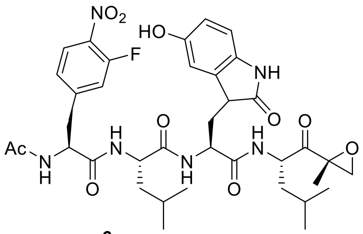

6

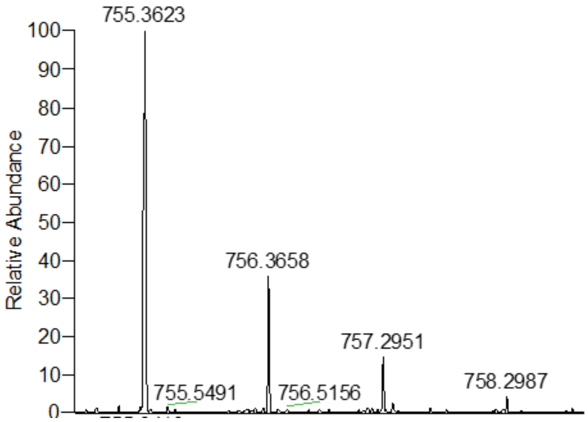

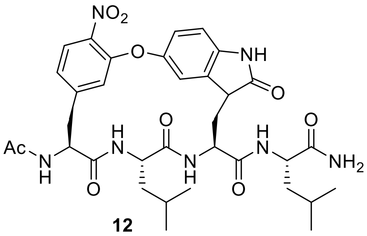

12

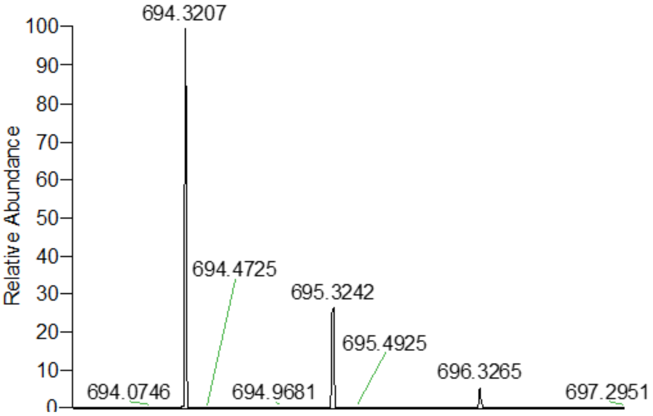

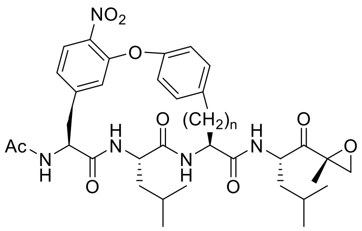

15a n=1

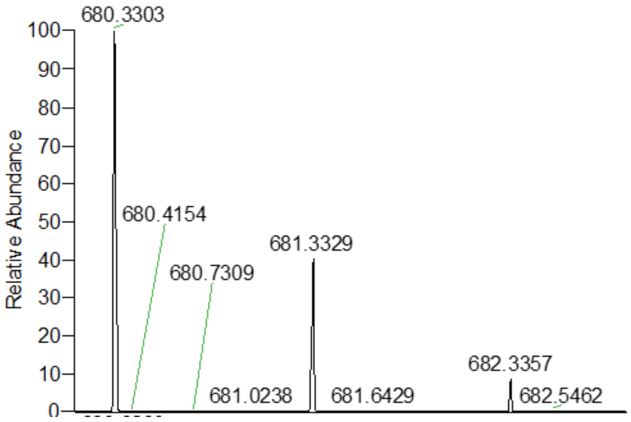
680.3303
680.4154
680.7309
681.3329
681.0238
681.6429
682.3357
682.5462
Relative Abundance
100
90
80
70
60
50
40
30
20
10
0

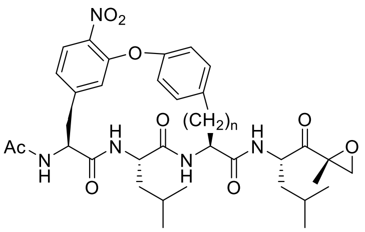

15b n=2

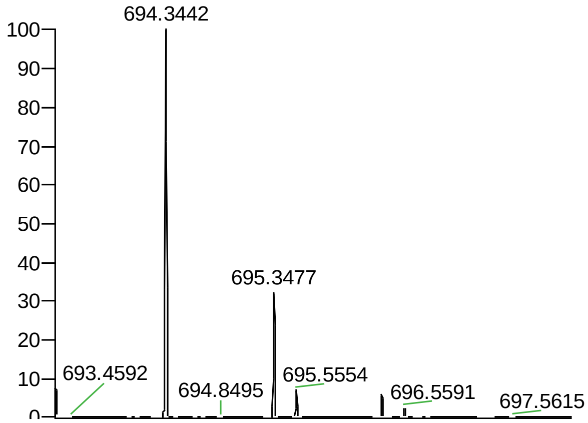
694.3442
695.3477
693.4592
694.8495
695.5554
696.5591
697.5615
100
90
80
70
60
50
40
30
20
10
0

5. COSY and LCMS (ESI) Spectra for Macrocyclic Epoxyketone 5

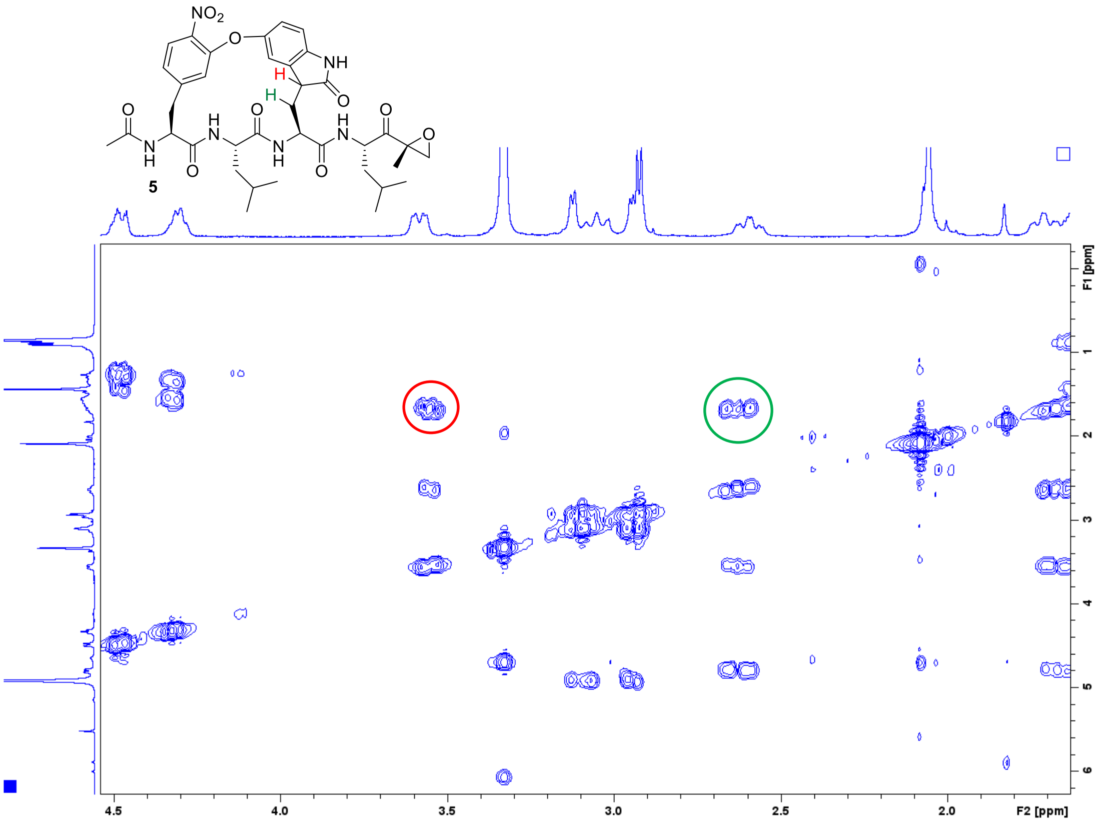

Figure 1S. COSY spectrum that identifies oxindole proton (red) based on spin-spin coupling to adjacent oxo-tryptophan proton (green).

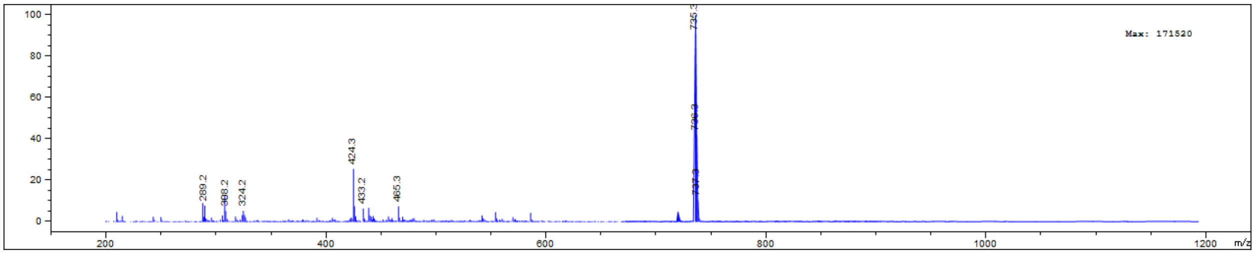

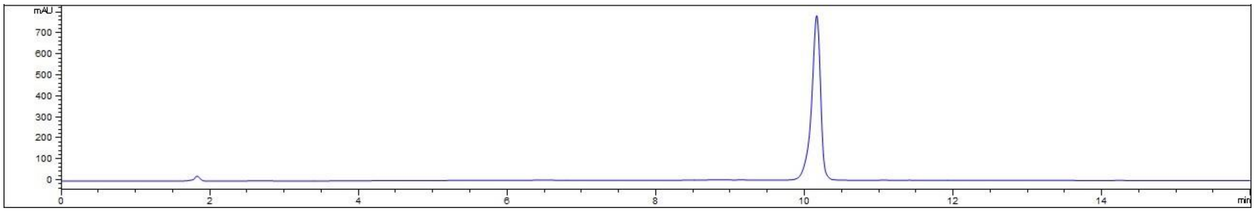

6. *In Vitro* Kinetic Enzyme Assays

The inhibitory potency and selectivity of the final compounds were tested using *in vitro* fluorescent enzyme kinetics. The enzyme assays used the fluorogenic substrate Suc-Leu-Leu-Val-Tyr-AMC (Suc-LLVY-AMC) and either the human 20S proteasome or alternate proteases chymotrypsin, m-calpain and cathepsin B. For cathepsin B, Cbz-Arg-Arg-AMC was used as the fluorogenic substrate. Kinetic enzyme assays were carried out on a fluorescent 96-well plate reader and AMC-substrate hydrolysis was measured at 353 nm excitation and 442 nm emission. Wells with inhibitor and wells with control solution were run in tandem. V_i and v_0 were calculated from the slope of the measured relative fluorescent units (RFUs) graphed against time for inhibitor and control wells, respectively. Values for v_i/v_0 were plotted as a function of inhibitor concentration with GraphPad Prism and IC_{50} values were calculated from a nonlinear regression analysis of the data ([Inhibitor] vs. response, three parameters).

Human 20S Proteasome ChT-L Activity: A well was loaded with 86 μL of buffer (20 mM HEPES, 0.5 mM EDTA, 0.037% SDS, pH 7.8), 2 μL of fluorogenic substrate (Suc-LLVY-AMC, 5 mM in DMSO), 20 μL of human 20S proteasome (0.34 μM in H_2O) and either 2 μL of inhibitor (at varying concentrations in DMSO) or 2 μL of DMSO (control). The fluorescent signal of the cleaved AMC chromophore was measured in RFUs at 37 °C over a span of 10 minutes with measurements taken every 20 seconds.

Human 20S Proteasome T-L Activity: A well was loaded with 69 μL of buffer (50 mM Tris, 1 mM EDTA, 100 mM NaCl, pH 7.5), 2 μL of fluorogenic substrate (Ac-RLR-AMC, 59 mM in DMSO), 2 μL of human 20S proteasome (2.6 μM in H_2O) and either 2 μL of inhibitor (at varying concentrations in DMSO) or 2 μL of DMSO (control). The fluorescent signal of the cleaved AMC chromophore was measured in RFUs at 37 °C over a span of 10 minutes with measurements taken every 20 seconds.

Human 20S Proteasome PGPH-L Activity: A well was loaded with 69 μL of buffer (50 mM Tris, 1 mM EDTA, 100 mM NaCl, pH 7.5), 2 μL of fluorogenic substrate (Cbz-LLE-AMC, 25 mM in DMSO), 2 μL of human 20S proteasome (2.6 μM in H_2O) and either 2 μL of inhibitor (at varying

concentrations in DMSO) or 2 μL of DMSO (control). The fluorescent signal of the cleaved AMC chromophore was measured in RFUs at 37 °C over a span of 10 minutes with measurements taken every 20 seconds.

Chymotrypsin: A well was loaded with 100 μL of buffer (0.05 M NaH_2PO_4/Na_2HPO_4, pH 7.5), 2 μL of fluorogenic substrate (Suc-LLVY-AMC, 5 mM in DMSO), 2 μL of chymotrypsin enzyme (3.33 μg/μL buffer) and either 2 μL of inhibitor (at varying concentrations in DMSO) or 2 μL of DMSO (control). The fluorescent signal of the cleaved AMC chromophore was measured in RFUs at 37 °C over a span of 10 minutes with measurements taken every 20 seconds.

Cathepsin B: A well was loaded with 100 μL of buffer [by volume: 95% kinetic buffer (0.1 M potassium phosphate, 1.25 mM EDTA, 0.01% Brij-35, pH 6.0), 1% DTT, 4% cathepsin B enzyme from human liver (0.441 μg/μL)], 2 μL of fluorogenic substrate (Cbz-Arg-Arg-AMC, 5 mM in DMSO), and either 2 μL of inhibitor (at varying concentrations in DMSO) or 2 μL of DMSO (control). The fluorescent signal of the cleaved AMC chromophore was measured in RFUs at 37 °C over a span of 10 minutes with measurements taken every 20 seconds.

m-Calpain: A well was loaded with 100 μL of kinetic buffer [by volume: 96.7% calpain buffer (50 mM HEPES, pH 7.5), 1.1% $CaCl_2$ (0.5 M in H_2O), 2.2% cysteine (0.5 mM in H_2O)], 2 μL of fluorogenic substrate (Suc-LLVY-AMC, 5 mM in DMSO), 2 μL of m-calpain enzyme (0.051 μg/μL kinetic buffer), and either 2 μL of inhibitor (at varying concentrations in DMSO) or 2 μL of DMSO (control). The fluorescent signal of the cleaved AMC chromophore was measured in RFUs at 37 °C over a span of 10 minutes with measurements taken every 20 seconds.

7. Crystallographic data collection

Crystallisation and structure determination of the yeast 20S proteasome core particle (yCP) in complex with compounds 5, 6, and 15a. Crystals of yCP were grown in hanging drops at 20 °C as previously described.[3] The protein concentration used for crystallization was 40 mg/mL in Tris / HCl (20 mM, pH 7.5) and EDTA (1 mM). The drops contained 1 µL of protein and 1 µL of the reservoir solution [30 mM magnesium acetate, 100 mM 2-(N-morpholino)ethanesulfonic acid (pH 6.8) and 10% (wt/vol) 2-methyl-2,4-pentanediol]. Crystals appeared after two days and were incubated with the respective ligand at final concentrations of 10 mM for at least 24 h. Droplets were then complemented with a cryoprotecting buffer [30% (wt/vol) 2-methyl-2,4-pentanediol, 15 mM magnesium acetate, 100 mM 2-(N-morpholino)ethanesulfonic acid, pH 6.9] and vitrified in liquid nitrogen. Datasets were collected using synchrotron radiation (λ =1.0 Å) at the X06SA-beamline (Swiss Light Source, Villingen, Switzerland). X-ray intensities and data reduction were evaluated using the XDS program package (Table 1S).[4] Conventional crystallographic rigid body, positional, and temperature factor refinements were carried out with REFMAC5[5] using coordinates of the yCP structure as starting model (PDB ID 5CZ4).[6] For model building, the programs SYBYL and COOT[7] were used. The final coordinates yielded excellent R factors, as well as geometric bond and angle values. Coordinates were confirmed to fulfill the Ramachandran plot and have been deposited in the RCSB.

Supporting Table S1. Crystallographic data collection and refinement statistics.

	yCP:5	***yCP:6***	***yCP:15a***
**Crystal parameters**			
Space group	$P2_1$	$P2_1$	$P2_1$
Cell constants	a = 135.7 Å	a = 135.7 Å	a = 135.2 Å
	b = 302.0 Å	b = 302.0 Å	b = 303.1 Å
	c = 144.2 Å	c = 144.2 Å	c = 143.2 Å
	β = 113.2 °	β = 113.2 °	β = 113.1 °
CPs / AU[a]	1	1	1
**Data collection**			
Beam line	X06SA, SLS	X06SA, SLS	X06SA, SLS
Wavelength (Å)	1.0	1.0	1.0
Resolution range (Å)[b]	30-3.05 (3.15-3.05)	30-3.05 (3.15-3.05)	30-3.2 (3.3-3.2)
No. observations	610989	610989	532525
No. unique reflections[c]	194819	194819	169944
Completeness (%)[b]	96.3 (98.4)	96.3 (98.4)	97.6 (98.3)
R_{merge} (%)[b, d]	9.2 (58.3)	9.2 (58.3)	9.1 (56.8)
I/σ (I)[b]	11.8 (2.6)	11.8 (2.6)	10.2 (2.2)
**Refinement (REFMAC5)**			
Resolution range (Å)	30-3.05	30-3.05	30-3.2
No. refl. working set	184936	184936	161289
No. refl. test set	9733	9733	8489
No. non hydrogen	49793	49793	49523
No. of ligand atoms	318	318	196
Solvent (H_2O, ions, MES)	171	171	91
R_{work}/R_{free} (%)[e]	17.6 / 21.2	17.6 / 21.2	18.4 / 22.8
r.m.s.d. bond (Å) / angle (°)[f]	0.003 / 1.2	0.003 / 1.2	0.002 / 1.2
Average B-factor ($Å^2$)	75.2	75.2	83.4
Ramachandran Plot (%)[g]	97.6 / 2.1 / 0.3	97.6 / 2.1 / 0.3	97.4 / 2.3 / 0.3
PDB accession code	8RHJ	8RHK	8RHL

[a] Asymmetric unit

[b] The values in parentheses for resolution range, completeness, R_{merge} and I/σ (I) correspond to the highest resolution shell

[c] Data reduction was carried out from a single crystal. Friedel pairs were treated as identical reflections

[d] $R_{merge}(I) = \Sigma_{hkl}\Sigma_j \mid I(hkl)_j - \langle I(hkl)\rangle \mid / \Sigma_{hkl} \Sigma_j I(hkl)_j$, where $I(hkl)_j$ is the jth measurement of the intensity of reflection hkl and <I(hkl)> is the average intensity

[e] $R = \Sigma_{hkl} \mid |F_{obs}| - |F_{calc}| \mid / \Sigma_{hkl} |F_{obs}|$, where R_{free} is calculated without a sigma cut off for a randomly chosen 5% of reflections, which were not used for structure refinement, and R_{work} is calculated for the remaining reflections

[f] Deviations from ideal bond lengths/angles

[g] Percentage of residues in favored / allowed / outlier region

8. References

[1] D. L. Wilson, I. Meininger, Z. Strater, S. Steiner, F. Tomlin, J. Wu, H. Jamali, D. Krappmann, M. G. Götz, *ACS Med. Chem. Lett.* **2016**, *7*, 250-255.

[2] D. L. Boger, R. M. Borzilleri, S. Nukui, R. T. Beresis, *J. Org. Chem.* **1997**, *62*, 4721-4736.

[3] a) M. Groll, R. Huber, *Methods Enzymol.* **2005**, *398*, 329-336; b) N. Gallastegui, M. Groll, *Methods Mol. Biol.* **2012**, *832*, 373-390.

[4] W. Kabsch, *Acta Crystallogr. D. Biol. Crystallogr.* **2010**, *66*, 125-132.

[5] G. N. Murshudov, P. Skubak, A. A. Lebedev, N. S. Pannu, R. A. Steiner, R. A. Nicholls, M. D. Winn, F. Long, A. A. Vagin, *Acta Crystallogr. D. Biol. Crystallogr.* **2011**, *67*, 355-367.

[6] E. M. Huber, W. Heinemeyer, X. Li, C. S. Arendt, M. Hochstrasser, M. Groll, *Nat. Com.* **2016**, *7*, 10900.

[7] P. Emsley, B. Lohkamp, W. G. Scott, K. Cowtan, *Acta Crystallogr. D. Biol. Crystallogr.* **2010**, *66*, 486-501.
